# Deep Learning for fODF Estimation in Infant Brains: Model Comparison, Ground‐Truth Impact, and Domain Shift Mitigation

**DOI:** 10.1002/hbm.70367

**Published:** 2025-10-07

**Authors:** Rizhong Lin, Hamza Kebiri, Ali Gholipour, Yufei Chen, Jean‐Philippe Thiran, Davood Karimi, Meritxell Bach Cuadra

**Affiliations:** ^1^ Signal Processing Laboratory (LTS5) École Polytechnique Fédérale de Lausanne (EPFL) Lausanne Switzerland; ^2^ Department of Diagnostic and Interventional Radiology Lausanne University Hospital (CHUV) and University of Lausanne (UNIL) Lausanne Switzerland; ^3^ School of Computer Science and Technology Tongji University Shanghai China; ^4^ CIBM Center for Biomedical Imaging Switzerland; ^5^ Computational Radiology Laboratory, Department of Radiology Boston Children's Hospital and Harvard Medical School Boston Massachusetts USA; ^6^ Department of Radiological Sciences University of California Irvine Irvine California USA; ^7^ Department of Electrical Engineering and Computer Science University of California Irvine Irvine California USA

**Keywords:** constrained spherical deconvolution (CSD), deep learning, diffusion MRI, domain adaptation, domain shift, fiber orientation distribution function (fODF), infant, neonate

## Abstract

The accurate estimation of fiber orientation distribution functions (fODFs) in diffusion magnetic resonance imaging (MRI) is crucial for understanding early brain development and its potential disruptions. Although supervised deep learning (DL) models have shown promise in fODF estimation from neonatal diffusion MRI (dMRI) data, the out‐of‐domain (OOD) performance of these models remains largely unexplored, especially under diverse domain shift scenarios. This study evaluated the robustness of three state‐of‐the‐art DL architectures: multilayer perceptron (MLP), transformer, and U‐Net/convolutional neural network (CNN) on fODF predictions derived from dMRI data. Using 488 subjects from the developing Human Connectome Project (dHCP) and the Baby Connectome Project (BCP) datasets, we reconstructed reference fODFs from the full dMRI series using single‐shell three‐tissue constrained spherical deconvolution (SS3T‐CSD) and multi‐shell multi‐tissue CSD (MSMT‐CSD) to generate reference fODF reconstructions for model training, and systematically assessed the impact of age, scanner/protocol differences, and input dimensionality on model performance. Our findings reveal that U‐Net consistently outperformed other models when fewer diffusion gradient directions were used, particularly with the SS3T‐CSD‐derived ground truth, which showed superior performance in capturing crossing fibers. However, as the number of input diffusion gradient directions increased, MLP and the transformer‐based model exhibited steady gains in accuracy. Nevertheless, performance nearly plateaued from 28 to 45 input directions in all models. Age‐related domain shifts showed asymmetric patterns, being less pronounced in late developmental stages (late neonates, and babies), with SS3T‐CSD demonstrating greater robustness to variability compared to MSMT‐CSD. To address inter‐site domain shifts, we implemented two adaptation strategies: the Method of Moments (MoM) and fine‐tuning. Both strategies achieved significant improvements (p<0.05) in over 95% of tested configurations, with fine‐tuning consistently yielding superior results and U‐Net benefiting the most from increased target subjects. This study represents the first systematic evaluation of OOD settings in DL applications to fODF estimation, providing critical insights into model robustness and adaptation strategies for diverse clinical and research applications.

## Graphical abstract

We quantify domain‐shift impacts of three state‐of‐the‐art deep learning models for fiber orientation estimation in dMRI of neonatal and baby brains, across age, scanner, input variations, target output ground truths, and demonstrate how fine‐tuning and data harmonization strategies improve model robustness for clinical and research applications.

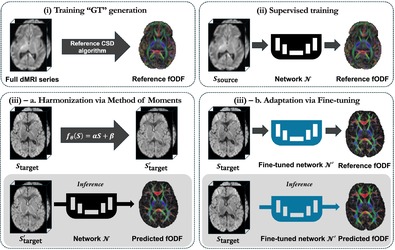

## 1 Introduction

Early brain development is a crucial period that sets the stage for lifelong health (Bhat et al. 2014; Godfrey and Barker 2001; O'Donnell and Meaney 2017; Volpe 2009). The depiction of white matter fiber bundles, which are responsible for relaying action potential signals between different brain areas, is of particular interest for fetal, newborn, and baby brains. Those long and myelinated axons have been shown to play a significant role in cognitive and motor functions from infancy (Dubois et al. 2014) to adulthood (Brun and Englund 1986; Davis et al. 2003; Ruiz‐Rizzo et al. 2024). Precise estimation of these bundles of fibers is essential to comprehend in vivo developmental trends and identify irregularities that might indicate potential diseases.

Progress in diffusion magnetic resonance imaging (dMRI), a noninvasive technique that relies on water molecule displacement as a proxy to microstructure, has yielded unparalleled insights into the mapping of the human brain (Descoteaux et al. 2011; Özarslan et al. 2013). The predominant method for extracting diffusion properties from the diffusion signal typically involves a prior model, commonly the diffusion tensor imaging (DTI) model (Basser et al. 1994). However, more intricate models such as multi‐shell multi‐tissue constrained spherical deconvolution (MSMT‐CSD) aim to reconstruct fiber orientation distribution functions (fODFs), enabling the representation of complex white matter configurations like fiber crossings (Jeurissen et al. 2014; Tournier et al. 2004) with sufficiently large crossing angles (Schilling et al. 2018). These models, however, necessitate densely sampled multi‐shell dMRI data that require high acquisition times. A less data‐demanding method, single‐shell three‐tissue constrained spherical deconvolution (SS3T‐CSD) (Dhollander and Connelly 2016; Dhollander et al. 2016), reconstructs multi‐tissue data with a single non‐zero *b*‐value and has been demonstrated to be a good fit for developing brains (Dhollander et al. 2019), where white matter voxels might suffer more from partial volume effect.

Imaging developing brains presents unique challenges that necessitate specialized approaches: Infants and young children have limited tolerance for long scan sessions, often requiring sedation or specialized acquisition protocols to minimize motion artifacts (Hughes et al. 2017). Motion artifacts are a primary concern in infant MRI scanning, as any head motion can degrade image quality and limit reliable assessment of brain structures (Coupe et al. 2013). These constraints necessitate faster acquisition times to guarantee sufficient data quality for reliable fiber orientation estimation.

### 1.1 Estimating fODFs With Machine Learning

With the advancement of machine learning techniques and more prominently deep learning, data‐driven approaches have emerged as powerful alternatives to traditional model‐based methods, enabling efficient estimation of the mapping between different related diffusion quantities, for instance, from dMRI signals to scalars such as fractional anisotropy from challenging or undersampled dMRI acquisitions (Alexander et al. 2014; Golkov et al. 2016; Karimi and Gholipour 2022; Tian et al. 2020). Also, fODF prediction from the original raw signal or its spherical harmonic (SH) representation has raised a growing interest from the community in recent years (Bartlett et al. 2023; Hosseini et al. 2022; Jha et al. 2023; Karimi, Vasung, et al. 2021; Kebiri, Gholipour, Lin, et al. 2023; Lin et al. 2019; Nath, Schilling, et al. 2019). Specifically, Nath, Schilling, et al. (2019) used a dense residual network to predict fODFs derived from ex vivo confocal microscopy images of monkey histology sections. However, this method is constrained by the unavailability of ex vivo histological training data. Although Karimi, Jaimes, et al. (2021) used a voxel‐wise multilayer perceptron (MLP) to predict fODFs, Bartlett et al. (2023) and Lin et al. (2019) employed a 3D convolutional neural network (CNN) to predict fODFs of the central voxel based on a small neighborhood of the diffusion signal. To further exploit the correlations among neighboring voxels, a two‐stage Transformer‐CNN was employed by Hosseini et al. (2022) to convert 200 measurements into 60 measurements before proceeding to predict fODFs. Another work (Jha et al. 2023) has shown, using a differential equation approach, the feasibility of predicting accurate fODFs with a limited number of diffusion gradient directions using a 2D neighborhood. On the other hand, Kebiri, Gholipour, Lin, et al. (2023) used a 3D U‐Net‐like network with extensive residual connections to predict big patches, leveraging spatial correlations to estimate fODFs with a small number of input measurements and hence a substantial reduction in scanning times. This approach has yielded promising results on newborns and fetuses (Kebiri et al. 2024).

Differently, Koppers and Merhof (2016) have tried to estimate the orientation of the fibers using 2D CNN in a classification paradigm. Other studies have aimed at segmenting fiber tracts either through a prior model applied on the input (Dong et al. 2019; Wasserthal et al. 2018) or directly from a spherical representation of the acquired signal (Kebiri, Gholipour, Bach Cuadra, and Karimi 2023). Additionally, Da Silva et al. (2024) and Zeng et al. (2022) have developed angular super‐resolution approaches to enhance fODF quality from limited single‐shell acquisitions (SS3T‐CSD) to approximate multi‐shell high angular reconstructions (MSMT‐CSD). An extensive review of machine learning applications in dMRI can be found in Karimi and Warfield (2024) and Kebiri (2023).

In contrast to these supervised approaches, unsupervised methods have emerged as an alternative paradigm for fODF estimation. These methods learn biophysical features directly from dMRI data, without requiring pre‐computed reference ground‐truth fODFs for training. Notable examples include equivariant spherical deconvolution methods (Elaldi et al. 2021, 2024, 2025), which use rotation‐equivariant neural networks to predict fODFs that are then convolved with tissue response functions to reconstruct the dMRI signal, optimizing based on signal reconstruction error. Implicit neural representations employ coordinate‐based neural networks to model continuous fODF fields, where the spatial encoding allows these models to learn spatial correlations between neighboring voxels rather than treating voxels independently: Consagra et al. (2024) specifically address how conventional approaches ignore valuable spatial correlations by using neural fields to parameterize spatially varying ODFs that implicitly model spatial correlation structures while incorporating uncertainty quantification, whereas Hendriks et al. (2025) adapt constrained spherical deconvolution (CSD) within an implicit neural framework using sinusoidal encoding for spatial regularization across multi‐shell data. More recently, Gao et al. (2026) proposed unsupervised three‐compartment learning that jointly estimates tissue fractions and fODFs by enforcing biophysical constraints in the optimization process. Although these unsupervised approaches offer the advantage of not requiring explicit training ground truth data, they present distinct considerations for developing brain imaging. These methods typically require dataset‐specific training phases, substantial computational resources for optimization, and often extensive acquisition protocols with high angular resolution data, which may pose challenges for clinical scenarios involving young subjects where rapid deployment and acquisition time constraints are critical. Furthermore, direct comparison between supervised and unsupervised methods remains methodologically complex and potentially unfair. Supervised approaches can be evaluated against established reference standards (e.g., CSD reconstructions), but using these same CSD‐derived ground truth labels to evaluate unsupervised methods would be inherently biased, as it would penalize methods that learn alternative, potentially superior representations of the underlying fiber architecture. The absence of truly independent in vivo ground truth (Karimi and Warfield 2024; Kebiri et al. 2024) further complicates fair comparative evaluation between these paradigms.

### 1.2 Domain Shifts in dMRI and Mitigation Strategies

Although DL applied in medical imaging offers strong advantages as detailed above, it suffers substantially from *domain shift* issues in which source and target data distributions vary considerably. In fact, small datasets that are limited in age span and the privacy constraints of sharing them at scale hamper cross‐dataset studies. In MRI (Richiardi et al. 2025), domain shift is even more amplified as scanners from different sites vary in brands and field strengths and sequence acquisition parameters that differ significantly, even within one modality such as dMRI (*b*‐values and the gradient directions as an example). Both biological shifts (Bento et al. 2022; Dubois et al. 2014), such as age or pathology, and technological shifts (Tax et al. 2019) contribute to the final distribution shift between the source sets and the target sets. Age is a particular shift in developing brains because of the rapid change in structure and function (Konkel 2018; Schilling et al. 2023).

To mitigate distribution shifts in MRI, solutions may operate at the data level (e.g., data harmonization or augmentation) or at the model level (e.g., transfer learning or domain adaptation).

Data harmonization, which aims to minimize differences due to the unwanted shift between the source domain and the target domain, is dominated by statistical methods (Cetin Karayumak et al. 2019; Huynh et al. 2019; Johnson et al. 2007; Karimi and Warfield 2024; Mirzaalian et al. 2018). The most dominant ones are the Rotation Invariant Spherical Harmonics (RISH) (Cetin Karayumak et al. 2019, 2024; Mirzaalian et al. 2018), specifically designed for the original dMRI signal, and combined association test (ComBat) (Fortin et al. 2017; Johnson et al. 2007), a more general harmonization method that is applied to the target diffusion map (Pinto et al. 2020), that is, after model fitting. The Method of Moments (MoM) (Huynh et al. 2019), which aligns diffusion‐weighted imaging (DWI) features via spherical moments, was also proposed recently and achieved promising results in developing brains (Lin, Gholipour, et al. 2024). While most harmonization methods, such as RISH, require similar acquisition protocols and site‐matched healthy controls, MoM and ComBat are not subject to these restrictions, making them appropriate for a broader range of conditions.

Deep learning has also been used for harmonizing dMRI metrics (Hansen et al. 2022; Koppers et al. 2019; Moyer et al. 2020; Nath, Remedios, et al. 2019a), including those for fODF estimation. However, they either need paired acquisitions with histology (Nath, Remedios, et al. 2019a, 2019b) or scan‐rescan (Yao, Rheault, et al. 2023; Yao et al. 2024) acquisitions. Although deep learning techniques offer solutions to nonlinear harmonization, they are prone to overfitting and require extensive training data, often from matched acquisitions, that are not easy to get (Bashyam et al. 2022; Pinto et al. 2020).

Domain adaptation methods have been employed to tackle domain shifts in medical imaging (Guan and Liu 2021). However, only two methods have been proposed in the context of dMRI, and they aim to tackle the diversity of dMRI acquisitions and, in particular, the *b*‐value. Kamphenkel et al. (2018) circumvented the domain shift by using a diffusion kurtosis model to estimate missing input values in a breast cancer classification task, while Yao, Newlin, et al. (2023) used a dynamic head to learn the different shell configurations using spherical convolutions to predict fODFs. However, these methods do not offer robustness to other shifts such as scanner, age, or different protocols (except the *b*‐value).

Using deep learning, two orthogonal approaches are particularly interesting: adversarial training and transfer learning (fine‐tuning). The former relies on learning invariant features through a domain‐agnostic loss function (Ganin et al. 2016; Kamnitsas et al. 2017), whereas the latter relies on pre‐trained weights from a target‐related dataset (Ghafoorian et al. 2017; Samala et al. 2018) that can range from a source dataset to a public dataset of natural images (Krizhevsky et al. 2012).

These domain adaptation approaches are designed for supervised methods (Guan and Liu 2021). For unsupervised learning approaches, domain shifts are theoretically less problematic, as they learn biophysical features and perform fODF prediction on the same dataset without requiring external training data, which creates data distribution shifts (Karimi and Warfield 2024).

### 1.3 Contributions of This Work

The exploration of fODF estimation under domain shifts remains limited. While Karimi, Vasung, et al. (2021) and Kebiri, Gholipour, Lin, et al. (2023) have extensively tested their fODF prediction models on developing neonatal brains, only Kebiri, Gholipour, Lin, et al. (2023) have investigated out‐of‐domain (OOD) performance. However, this OOD evaluation was conducted qualitatively due to the lack of fetal fODF ground truth. Our preliminary work in Lin, Gholipour, et al. (2024) explored the age‐ and age/scanner/protocol‐related domain shifts and proposed potential solutions, including the MOM (data harmonization) and fine‐tuning (domain adaptation).

In this study, we significantly extend (Lin, Gholipour, et al. 2024) by including three state‐of‐the‐art supervised deep learning models: MLP‐based, transformers‐based, and U‐Net/CNN‐based. We also use two cohorts: the newborns of the developing Human Connectome Project (dHCP) and the babies of the Baby Connectome Project (BCP). We extend our analysis to different fODF *ground‐truth* models: SS3T‐CSD (Dhollander and Connelly 2016; Dhollander et al. 2016) and MSMT‐CSD (Jeurissen et al. 2014). Furthermore, different methodological configurations are evaluated: the number of input diffusion gradient directions to the model and the number of subjects from the target set used for domain adaptation/harmonization. To the best of our knowledge, this is the first study to assess OOD settings of DL applications for fODF estimation thoroughly.

## 2 Methods

**FIGURE 1:**
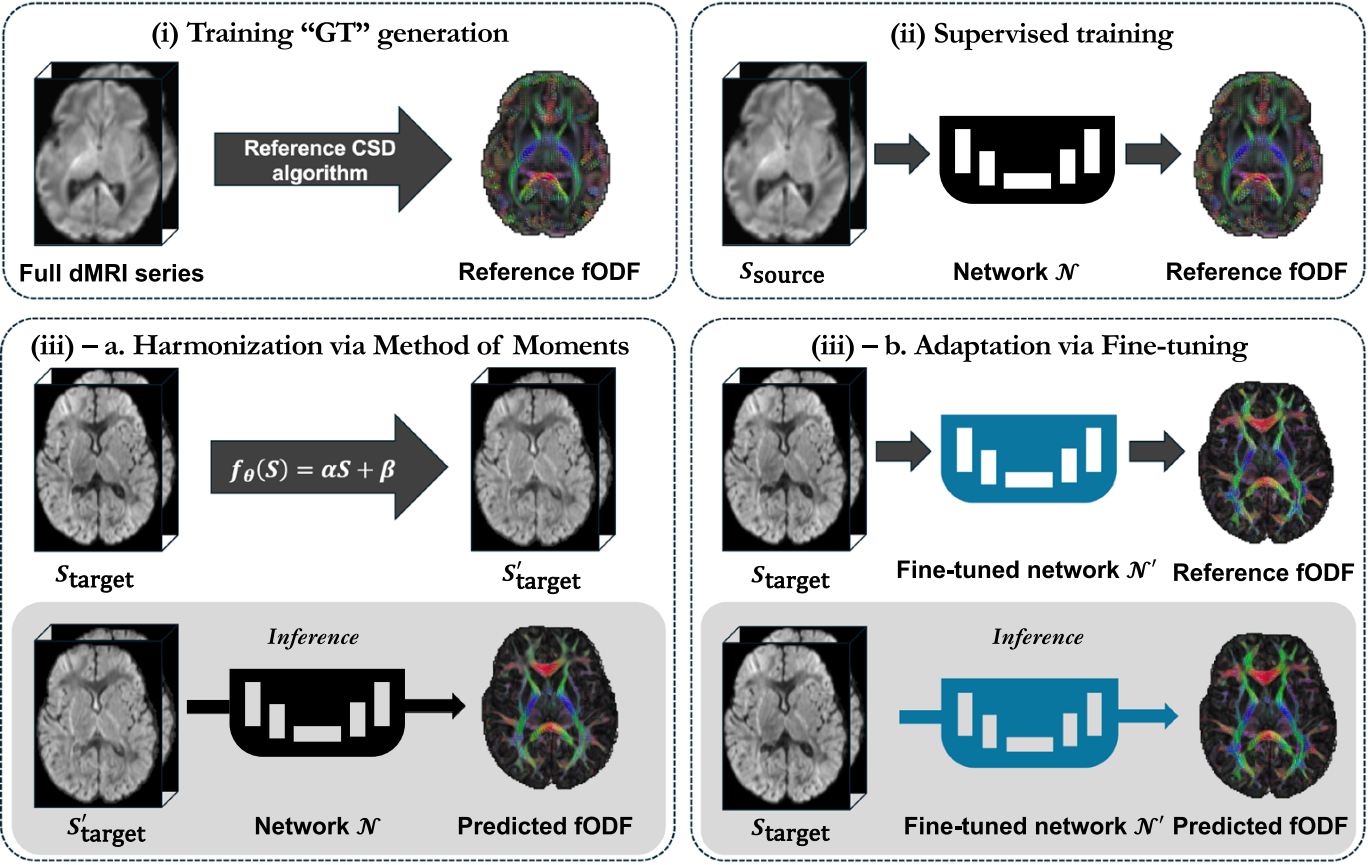
Overview of the fODF prediction framework using multiple deep learning architectures $N\in \left\{\mathrm{MLP},\text{CTtrack},U‐\mathrm{Net}\right\}$. (i) Reference fODFs are generated from full dMRI series using CSD algorithms (MSMT‐CSD or SS3T‐CSD) as ground truth for training of deep learning networks. (ii) Models are trained on source domain data ($S_{\text{source}}$) to predict these reference fODFs. (iii) Domain shifts (both intra‐site and inter‐site) are addressed through either: (a) MOM harmonization ($f_{\theta }\left(S\right)=\mathrm{\alpha S}+\beta$) to transform target domain data ($S_{\text{target}}$) before inference with the original model N, or (b) Fine‐tuning the original model N on target domain data to create an adapted model $N^{\prime }$. Here, $S_{\text{source}}$ denotes the source domain dataset, $S_{\text{target}}$ denotes the target domain dataset, and $N^{\prime }$ represents the fine‐tuned model. The framework enables comprehensive evaluation across different acquisition protocols and age ranges, as detailed in Section 3.

Our framework for fODF prediction and OOD evaluation is illustrated in Figure 1 in multiple stages. First, reference CSD algorithms (MSMT‐CSD, SS3T‐CSD) generate ground truth fODFs from full dMRI series for training. Next, deep learning models $N\in \left\{\mathrm{MLP},\text{CTtrack},U‐\mathrm{Net}\right\}$ undergo supervised training using source domain data ($S_{\text{source}}$) to predict these reference fODFs. The framework is evaluated in both intra‐site and inter‐site scenarios (detailed in Section 3), with domain shifts addressed through either data harmonization using MOM to transform target domain data ($S_{\text{target}}$) before inference, or model adaptation via fine‐tuning on target domain data to create adapted networks ($N^{\prime }$).

### 2.1 Backbone Model Architectures

Three distinct deep learning architectures were implemented for fODF prediction, each representing different modeling paradigms: a U‐Net‐based architecture for multiscale spatial context learning (Kebiri, Gholipour, Lin, et al. 2023), a hybrid CNN‐Transformer for local–global feature integration (Hosseini et al. 2022), and an MLP for direct voxel‐wise signal‐to‐fODF mapping (Karimi, Vasung, et al. 2021). These architectures share a common input–output framework: Input diffusion measurements are normalized and projected onto a SHs basis to enhance acquisition independence, with SH order selection determined by the number of input directions. Throughout this paper, $n_{\mathrm{sig}}$ denotes the number of input diffusion measurements, whereas the output consistently represents fODF in SH basis (SH‐*L*_max_ order 8) with 45 coefficients, denoted as $n_{\mathrm{sh}}=45$.

#### 2.1.1 U‐Net Based Method

The U‐Net architecture (Kebiri, Gholipour, Lin, et al. 2023) extends the traditional U‐Net (Ronneberger et al. 2015) with extensive multiscale residual connections and hierarchical feature integration. The network processes $16\times 16\times 16\times n_{\mathrm{sig}}$ patches through an encoder‐decoder structure with configurable depth. The encoder uses stride‐2 convolutions for downsampling, with feature maps doubling at each level starting from 36 channels. Each encoding level incorporates residual blocks and cross‐scale connections that concatenate features from different resolution levels. The decoder employs transposed convolutions for upsampling, concatenating encoder features via skip connections before applying residual blocks. All convolutional blocks use ReLU activation (Agarap 2018) and dropout (Srivastava et al. 2014) for regularization, with normalization applied in activation‐dropout‐normalization ordering. The output layer produces $16\times 16\times 16\times n_{\mathrm{sh}}$ fODF predictions without activation to enable direct SH coefficient regression.

#### 2.1.2 CNN‐Transformer (CTtrack)

This hybrid architecture (Hosseini et al. 2022) combines 3D CNNs for spatial feature extraction with Transformer networks for global context modeling. The network processes $3\times 3\times 3\times n_{\mathrm{sig}}$ patches through a residual CNN block with 60 feature maps and GELU activation (Hendrycks and Gimpel 2016). The CNN output is divided into 27 spatial patches, each projected to a *d*‐dimensional embedding space and augmented with learned positional encodings. Four Transformer blocks with multi‐head self‐attention (Vaswani et al. 2017) and feed‐forward networks process these patch embeddings, using layer normalization (Ba et al. 2016) and residual connections (He et al. 2016). Global average pooling aggregates the attention features, which are then processed by an MLP head with GELU activation to predict $n_{\mathrm{sh}}=45$ SH coefficients for the central voxel.

#### 2.1.3 Multilayer Perceptron (MLP)

The MLP architecture (Karimi, Vasung, et al. 2021) operates on a voxel‐wise basis, processing individual voxels independently without spatial neighborhood information. The network comprises an input layer, six hidden layers, and an output layer with neuron configuration $\left[n_{\mathrm{sig}},300,300,300,400,500,600,n_{\mathrm{sh}}\right]$, where $n_{\mathrm{sig}}$ represents the number of input diffusion measurements and $n_{\mathrm{sh}}=45$ corresponds to the SH coefficients of the output fODF. Hidden layers use ReLU activation functions (Agarap 2018) with variance scaling initialization (He et al. 2015), while the output layer has no activation function to enable direct regression of SH coefficients. Dropout layers (Srivastava et al. 2014) are applied after each dense layer for regularization. The fully connected architecture enables direct signal‐to‐fODF mapping without spatial context considerations.

### 2.2 Reference CSD‐Based fODF Reconstruction for Training

To generate *training ground truths*, we used two classical fODF estimation models, Multi‐Shell Multi‐Tissue CSD (MSMT‐CSD) (Jeurissen et al. 2014) and Single‐Shell 3‐Tissue CSD (SS3T‐CSD) (Dhollander and Connelly 2016) under various dMRI acquisition settings. Following established terminology (Lin, Kebiri, et al. 2024), we refer to these fODF reconstructions as “ground truth” or “GT” when used as training targets for the deep learning models, while acknowledging these are reference reconstructions rather than true anatomical measurements. MSMT‐CSD leverages multi‐shell data to compute tissue‐specific response functions and yields multi‐tissue fODFs. SS3T‐CSD uses b1000 single‐shell (+b0) data and a three‐tissue approach, enabling approximate multi‐tissue decomposition with reduced acquisition demands. These reconstructions from classical models serve as training targets for the deep learning models.

### 2.3 Training Details

The U‐Net training employed Adam optimizer (Kingma and Ba 2014) with $\ell_{2}$ norm loss, initial learning rate $2\times 10^{-4}$, 0.1 dropout rate, and batch size 1, processing 128 patches per epoch for 500–1000 epochs with early stopping. CTtrack training used AdamW optimizer (Loshchilov and Hutter 2019) with initial learning rate $1\times 10^{-3}$, weight decay $5\times 10^{-3}$, batch size 4000, and $\ell_{1}$ loss function for 100 epochs. The MLP employed Adam optimizer with initial learning rate $1\times 10^{-3}$, batch size 2000, and $\ell_{2}$ norm loss for 100 epochs. The architectures have varying computational complexity: the MLP contains approximately 0.8 M parameters (for $n_{\mathrm{sig}}=45$), CTtrack contains approximately 1.8 M parameters, and U‐Net contains approximately 7 M parameters. All models demonstrated stable convergence behavior, with training loss consistently decreasing and validation loss reaching a plateau within the specified epochs, indicating successful optimization without overfitting. Convergence times approximated 12, 12, and 4 h for U‐Net, CTtrack, and MLP, respectively, with inference requiring less than 10 s per subject.

### 2.4 Domain Shift Mitigation Methods

We evaluate two domain adaptation strategies: data harmonization using MoM and model adaptation through fine‐tuning. These approaches address domain shifts through fundamentally different mechanisms and have distinct data requirements.

#### 2.4.1 Data Harmonization Based on MoM

We used the MoM approach (Huynh et al. 2019) to harmonize dMRI data across different sites or acquisition protocols, as it is not subject to protocol‐ or subject‐matched constraints. This approach requires only the DWI signals from target domain subjects, without requiring corresponding ground truth fODFs. A linear transformation:

(1) $$f_{\theta }\left(S\right)=\mathrm{\alpha S}+\beta$$

matches the statistical moments of the source and target datasets. For each gradient direction, we compute the mean ($m_{1}$) and variance ($m_{2}$) of the target and source data within brain masks. Voxel‐wise estimates of α and β are obtained by minimizing:

(2) $$\text{cost}={\left(m_{1,\text{source}}-\left(\alpha \;m_{1,\text{target}}+\beta \right)\right)}^{2}+\lambda_{1}\;R\left(\alpha ,\beta \right)+\lambda_{2}\;{\left(m_{2,\text{source}}-\alpha^{2}\;m_{2,\text{target}}\right)}^{2}$$

where $R\left(\alpha ,\beta \right)$ is a regularization term penalizing large deviations from identity:

(3) $$R\left(\alpha ,\beta \right)=\frac{1-N\left(\alpha |1,1\right)}{N\left(1|1,1\right)}+\frac{1-N\left(\beta |0,1\right)}{N\left(0|0,1\right)}$$

This voxel‐wise linear optimization is computationally efficient, requiring only seconds to minutes for harmonization of an entire dataset.

#### 2.4.2 Domain Adaptation by Fine‐Tuning

The domain adaptation framework employs transfer learning through model fine‐tuning, maintaining the same architecture while adjusting network parameters to the target domain data distribution. In contrast to MoM harmonization, fine‐tuning requires both DWI signals and corresponding ground truth fODFs from target domain subjects for supervised learning.

Each model maintained its original optimizer while adapting to target domain data. The U‐Net processed target data in $16^{3}$ patches with stride‐8 voxels (50% overlap in each dimension), using only patches within the brain mask. These patches were split into training and validation sets (4:1 ratio). Fine‐tuning ran for 20 epochs with learning rate $1\times 10^{-5}$ and batch size 64 for U‐Net, while CTtrack and MLP used 10 epochs with learning rates $1\times 10^{-4}$ and $2\times 10^{-4}$, respectively, and batch size 2000. Fine‐tuning computational time per epoch varied with the number of target subjects and brain size: approximately 10 s (1 subject) to 60 s (10 subjects) per epoch for U‐Net, 30–150 s for CTtrack, and 3–15 s for MLP, with BCP subjects requiring modestly longer processing times due to larger brain and white matter volumes.

### 2.5 Statistical Analysis

Statistical significance was assessed using nonparametric tests appropriate for neuroimaging performance metrics, which typically exhibit bounded distributions and non‐normal characteristics (Button et al. 2013). Wilcoxon signed–rank tests (Wilcoxon 1945) were used for paired comparisons involving the same test subjects under different conditions. Mann–Whitney *U* tests (Mann and Whitney 1947) were employed for unpaired comparisons between distinct subject populations. All tests were two‐tailed unless directional hypotheses were being tested, such as performance improvement with increased gradient directions. Bonferroni correction (Dunn 1961) was applied to control for multiple comparisons within each experimental analysis. Tests were categorized by experimental design: intra‐site performance comparisons, age‐related domain shift analyses, and inter‐site domain adaptation assessments, with method‐to‐method comparisons providing direct architecture validation.

### 2.6 Implementation Details

All models were implemented using PyTorch (Paszke et al. 2019), PyTorch Lightning (Falcon and The PyTorch Lightning team 2024), MONAI (Cardoso et al. 2022), and TensorFlow 2 (Abadi et al. 2015). The U‐Net architecture was reimplemented from its original TensorFlow version using PyTorch. CTtrack maintained its original TensorFlow 2 implementation with modified data loading pipelines. The MLP architecture was reconstructed in TensorFlow 2 following published specifications. The MoM harmonization was implemented in MATLAB R2022b. Training used an NVIDIA GeForce RTX 2080 Ti with 11GB RAM and 24 CPU cores. All statistical analyses were performed using SciPy (Virtanen et al. 2020).

### 2.7 Evaluation

A quantitative assessment was carried out to evaluate the performance of the fODF estimation methods. The evaluation process relied on five metrics that capture different aspects of fODF reconstruction quality: (1) peak‐based metrics (Kebiri et al. 2024), including agreement rates (ARs) and angular differences (AD) that assess fiber population detection accuracy for both single and crossing fiber configurations, (2) fODF‐derived microstructural measures, including apparent fiber density (AFD) (Raffelt et al. 2012), which quantifies signal amplitude, and generalized fractional anisotropy (GFA) (Tuch 2004), which measures directional coherence and shape anisotropy, and (3) global correlation measures using the angular correlation coefficient (ACC) (Anderson 2005), which evaluate overall fODF field consistency and spatial coherence. Together, these metrics provide a comprehensive evaluation across multiple dimensions of fODF reconstruction quality. For peak‐based analysis, fiber orientations were extracted using Dipy (Garyfallidis et al. 2014) with a mean separation angle of 45°, a maximum of 3 peaks, and a relative peak threshold of 0.5. The choice of these parameters was guided by the work of Schilling et al. (2018), which demonstrated the limitations of current dMRI models in correctly estimating multiple fiber populations and low angular crossing fibers.

Agreement rate The AR was computed using confusion matrices and defined for each number of peaks p as:

(4) $$\text{Agreement rate}=\frac{A_{p}}{\sum D_{p}}$$

where $A_{p}$ represents the percentage of voxels where both methods agree on p number of peaks, and $D_{p}$ denotes the percentage of voxels where at least one of the two methods predicts p peaks. This metric captures the rate of concordance between two methods in peak number estimation.

Angular difference: Mean angular difference was computed for voxels containing the same number of estimated peaks. For voxels with multiple fibers, we extracted corresponding peaks between methods by computing the minimum angle between all possible configurations (4 configurations for 2 peaks, 9 for 3 peaks), followed by recursive elimination of matched peaks until all peaks are paired. When comparing deep learning predictions against training ground truth, this metric represents angular error; when comparing split datasets or different reconstruction methods, it quantifies angular disagreement.

Apparent fiber density: The AFD (Raffelt et al. 2012) quantifies the density of fibers aligned in specific orientations by integrating the fODF within directionally‐coherent lobes. For a given fODF $f\left(u\right)$, the AFD is computed by first segmenting the fODF into lobes based on peaks and troughs, then calculating the integral over each lobe:

(5) $${\mathrm{AFD}}_{\text{lobe}}=\int_{\text{lobe}}\;f\left(u\right)\;du$$

where the integration is performed over each segmented lobe region of the fODF on the unit sphere. This approach captures the total fiber density within each coherent fiber population rather than just peak amplitude. The AFD difference is computed as a masked mean absolute percentage difference (MAPD), where the mask is confined to white matter voxels, and is given by:

(6) $$\mathrm{AFD}\;\text{difference}=\frac{100}{n}\sum_{i\in \mathrm{WM}}\left|\frac{{\mathrm{AFD}}_{1,i}-{\mathrm{AFD}}_{2,i}}{{\mathrm{AFD}}_{2,i}}\right|$$

where n is the number of white matter voxels, and ${\mathrm{AFD}}_{1,i}$ and ${\mathrm{AFD}}_{2,i}$ represent the AFD values from the two methods being compared at voxel i. This formulation provides a unified metric that serves as an error measure when evaluating predictions against ground truth and as a disagreement measure when comparing equivalent reconstructions.

Generalized fractional anisotropy: The GFA (Tuch 2004) is a scalar measure that quantifies the normalized variance of the fODF, indicating the degree of anisotropy. For an fODF with discrete samples $f=\left[f_{1},f_{2},\ldots ,f_{N}\right]$ on the sphere, the GFA is computed as:

(7) $$\mathrm{GFA}=\frac{\mathrm{std}\left(f\right)}{\mathrm{rms}\left(f\right)}=\frac{\sqrt{\frac{1}{N}\sum_{i=1}^{N}{\left(f_{i}-\bar{f}\right)}^{2}}}{\sqrt{\frac{1}{N}\sum_{i=1}^{N}f_{i}^{2}}}$$

where $\bar{f}=\frac{1}{N}\sum_{i=1}^{N}\;f_{i}$ is the mean value of the fODF samples, and N is the number of sampling directions on the sphere. GFA values range from 0 (isotropic, no directional preference) to 1 (maximally anisotropic, single direction). The GFA difference is computed as the absolute difference between GFA values within white matter voxels:

(8) $$\mathrm{GFA}\;\text{difference}=\frac{1}{n}\sum_{i\in \mathrm{WM}}\left|{\mathrm{GFA}}_{1,i}-{\mathrm{GFA}}_{2,i}\right|$$

where n is the number of white matter voxels. This provides a complementary measure to AFD that captures structural anisotropy characteristics rather than amplitude differences.

Angular correlation coefficient: The ACC (Anderson 2005) provides a global measure of similarity between fODF fields. For two fODFs represented by their SH coefficients $c^{\left(1\right)}$ and $c^{\left(2\right)}$, the ACC is computed as:

(9) $$\mathrm{ACC}=\frac{c^{\left(1\right)}\cdot c^{\left(2\right)}}{\left\|c^{\left(1\right)}\right\|\left\|c^{\left(2\right)}\right\|}$$

where $c^{\left(1\right)}\cdot c^{\left(2\right)}$ represents the dot product of the SH coefficient vectors, and $\left\|c\right\|$ denotes the Euclidean norm. This metric quantifies the overall correlation between fODF shapes and is particularly useful for assessing consistency between different reconstruction methods applied to the same underlying data.

## 3 Experiments

The proposed framework was extensively evaluated through a series of experiments designed to assess both the performance of different network architectures ($N\in \left\{\mathrm{MLP},\text{CTtrack},U‐\mathrm{Net}\right\}$) and their robustness to various domain shifts. We first validate the CSD‐based reconstruction methods used for ground truth generation, then investigate intra‐site domain shifts (particularly age‐related variations), and finally examine inter‐site domain shifts between two major developing brain imaging consortia. The effectiveness of our domain shift mitigation strategies is evaluated across these scenarios.

### 3.1 Datasets and Preprocessing

#### 3.1.1 Neonates of the dHCP

We use the third data release of the publicly available dHCP dataset^1^ (Edwards et al. 2022), which was acquired on a 3 T Philips Achieva system with a 32‐channel neonatal head coil. The protocol employed TE = 90 ms, TR = 3800 ms, a multiband factor of 4, a SENSE factor of 1.2, a Partial Fourier factor of 0.855, in‐plane resolution 1.5 mm^2^, slice thickness 3 mm with 1.5 mm overlap (Hutter et al. 2018), and four shells $\left\{0,400,1000,2600\right\}$ s/mm^2^ with 20, 64, 88, and 128 volumes. Data were preprocessed with SHARD (Christiaens et al. 2021; Pietsch et al. 2021), including MP‐PCA denoising (Veraart et al. 2016), motion and distortion correction, Gibbs suppression, and resampling to 1.5 mm^3^ isotropic resolution of size 100×100×64 voxels. White matter masks were obtained by combining T2‐weighted segmentation labels *White Matter* and *Brainstem* (registered to diffusion space Yushkevich et al. 2016) with FA >0.25 computed in MRtrix (Tournier et al. 2012), following Kebiri et al. (2024). We used a total of 323 unique subjects from dHCP. Of these, 165 subjects (PMA [postmenstrual age] at scan: $\left[35.57,45.29\right]$ weeks, median: 40.29, mean: 40.01, SD: 2.29) were denoted as dataset $S_{\text{dHCP}}$. Additionally, we selected two age‐distinct sets from dHCP, denoted as $S_{\text{dHCP},\text{early}}$ and $S_{\text{dHCP},\text{late}}$, respectively, each consisting of 105 subjects: early‐stage (PMA at scan: $\left[33.14,37.86\right]$ weeks, median: 35.57, mean: 35.65, SD: 1.39) and late‐stage (PMA at scan: $\left[41.0,45.14\right]$ weeks, median: 42.43, mean: 42.48, SD: 1.07).

#### 3.1.2 Babies of the Human Connectome Project (BCP)

We used the data from the publicly available BCP dataset^2^ (Howell et al. 2019). Images were acquired on a 3 T Siemens Magnetom Prisma with a 32‐channel head coil. The dMRI protocol used six shells $b\in \left\{500,1000,1500,2000,2500,3000\right\}$ s/mm^2^ having 9, 12, 17, 24, 34, 48 diffusion gradient directions, respectively, and 6 b0 images. Other parameters included TE = 88.6 ms, TR = 2640 ms, multiband factor 5, resolution 1.5 mm^3^, with a field of view of 140×140×96 voxels. Preprocessing included denoising, bias correction, motion compensation, and distortion correction (Andersson and Sotiropoulos 2016), followed by FSL BET brain extraction (Jenkinson et al. 2012). White matter masks were derived by combining a SynthSeg^+^ (Billot et al. 2023) WM mask, voxels with FA > 0.4, and voxels with (FA > 0.15) ∧ (MD > 0.0011). We used 165 subjects from BCP (age at scan: $\left[2.0,24.0\right]$ months, median: 15.00, mean: 15.91, SD: 5.87) and denote this dataset as $S_{\mathrm{BCP}}$.

### 3.2 Validation of Reference CSD‐Based Reconstruction Methods

To assess the quality and consistency of the reconstruction methods used to generate training targets, we split each subject's dMRI series into two equal, nonoverlapping subsets (GS1 and GS2), following Kebiri, Gholipour, Lin, et al. (2023) and Kebiri et al. (2024). To ensure optimal angular distribution and prevent potential bias, we employed a systematic splitting methodology that optimizes the condition number of the diffusion tensor reconstruction matrix, following the scheme proposed by Skare et al. 2000. The splitting process operated independently for each *b*‐value shell, selecting the directions that provide optimal condition number properties as GS1, whereas the remaining directions formed GS2. This approach ensures that both subsets represent meaningful sampling schemes with complementary angular coverage rather than arbitrary divisions.

For the dHCP dataset, each subset contained 150 measurements distributed across *b*‐values {0, 400, 1000, 2600} s/mm^2^ with 10, 32, 44, and 64 directions, respectively. Similarly, for the BCP dataset, each subset contained 75 measurements. We reconstructed fODFs using MSMT‐CSD on both datasets using all available *b*‐values. For the dHCP dataset, we additionally performed SS3T‐CSD reconstruction using only the b=0 and b=1000 s/mm^2^ shells.

As GS1 and GS2 represent equivalent samplings of the same underlying diffusion signal, the difference between their reconstructed fODFs indicates the inherent variability in each reconstruction method. This comparison quantifies the reference method consistency under reduced sampling conditions for classical CSD approaches when applied to subsampled data. For the dHCP dataset, we also compared the fODFs reconstructed from the complete series (300 measurements for MSMT‐CSD and b0/b1000 measurements for SS3T‐CSD) to assess and understand the differences between these two reconstruction approaches.

### 3.3 Intra‐Site Performance and Age‐Related Domain Shift

We evaluated within‐domain performance using both $S_{\text{dHCP}}$ and $S_{\mathrm{BCP}}$ datasets as defined in Section 3.1, each containing 165 subjects. The subjects were split into 85/80 for training‐validation/testing. Age‐related variations were investigated exclusively in the dHCP dataset, as BCP data showed minimal age‐related variation (Lin, Gholipour, et al. 2024). For age‐specific analysis, we used $S_{\text{dHCP},\text{early}}$ and $S_{\text{dHCP},\text{late}}$, containing 105 subjects each, split 50/55 for training‐validation/testing.

For input signals, we used varying numbers of directions ($n_{\mathrm{sig}}$): {6, 15, 28, 45} for dHCP and {6, 12} for BCP, with normalization by b0. The specific gradient directions for each $n_{\mathrm{sig}}$ were selected using the scheme proposed by Skare et al. 2000, which minimizes the condition number of the diffusion tensor reconstruction matrix. Ground truth fODFs were generated using MSMT‐CSD for both datasets. For dHCP, we conducted additional experiments using SS3T‐CSD‐generated fODFs from all 88 b1000 and 20 b0 measurements as an alternative training ground truth. For baseline comparisons, we performed in‐domain testing (training and testing on the same age group).

### 3.4 Inter‐Site Domain Shift

This analysis quantifies performance degradation when training on $S_{\text{dHCP}}$ but testing on $S_{\mathrm{BCP}}$ and vice versa. The mismatch includes different MRI scanners (Philips 3 T for dHCP, Siemens 3 T for BCP), acquisition protocols, and subject age ranges. We used 165 subjects from each dataset ($S_{\text{dHCP}}$ and $S_{\mathrm{BCP}}$), split into 85/80 for training‐validation/testing. Both datasets were normalized by b0, and we investigated $n_{\mathrm{sig}}\in \left\{6,12\right\}$ for each network, with gradient directions selected using the scheme proposed by Skare et al. 2000, comparing how domain shift impacts the fODF estimation results.

### 3.5 Domain Shift Mitigation

We evaluated two domain shift mitigation strategies when transferring models between the two sites (dHCP and BCP), as described in Section 2.4. Using {1, 2, 5, 10} target subjects, we tested both MoM harmonization, which transformed the input dMRI signals to match target domain statistics, and model fine‐tuning, which utilized both dMRI signals and ground truth fODFs to adapt the pre‐trained source model. As shown in Figure 1, we compared two inference scenarios: (1) applying the original model to harmonized target data and (2) using the fine‐tuned model on the original target data. These experiments quantified the effectiveness of each approach in improving fODF reconstruction quality across domains.

## 4 Results

We present our comprehensive evaluation results, with all performance differences validated through statistical testing: A total of 3633 statistical tests were performed across all experimental conditions, including 756 direct method‐to‐method comparisons for inter‐site experiments. These tests evaluated seven performance metrics (ARs for 1‐fiber and 2‐fiber voxels, ADs for 1‐fiber and 2‐fiber voxels, AFD, ACC, and GFA) across three CNN architectures, multiple gradient direction counts, and two ground truth reconstruction methods (MSMT‐CSD and SS3T‐CSD).

### 4.1 Consistency of Reference fODF Reconstructions

Table 1 shows the consistency between fODFs reconstructed from GS1 and GS2 of each subject using classical reconstruction methods across five complementary metrics. Single‐fiber estimations show a good AR (92% for dHCP and 86% for BCP) for MSMT‐CSD, while two‐fiber estimations exhibit lower ARs of around 45% for both datasets, with corresponding ADs of approximately $16^{{}^{\circ}}$. SS3T‐CSD exhibits higher agreement for multiple fibers (56%) and lower agreement for single fibers (63%). As reported previously (Lin, Kebiri, et al. 2024), the proportion of multiple fibers predicted by SS3T‐CSD (61%) is closer to literature values (66%) compared to MSMT‐CSD (23%) (Jeurissen et al. 2013; Schilling et al. 2022). Moreover, the ΔGS splitting results in each subset containing only 44 b1000 directions, which approaches the minimum number of gradient directions needed to adequately represent the 45 SH coefficients of an 8th‐order reconstruction (Tournier et al. 2013). Hence, increasing the amount of data available for generating the reference fODF reconstructions would likely improve the consistency between split‐dataset reconstructions.

**TABLE 1:** Consistency of reference fODF reconstructions.

Dataset	Model	$n_{m}$	Agreement rate (angular diff.)		AFD diff. (MAPD)	ACC	GFA diff.
			Single fiber	Two fiber			
dHCP	MSMT‐CSD	150	92.4% (6°)	44.2% (16°)	0.206±0.08	0.960±0.009	0.022±0.004
dHCP	SS3T‐CSD	54	62.7% (6°)	55.9% (13°)	0.066±0.01	0.916±0.017	0.032±0.003
BCP	MSMT‐CSD	75	86.0% (6°)	45.5% (17°)	0.117±0.05	0.927±0.019	0.028±0.003

*Note:* We compare fODFs generated from two nonoverlapping subsets of each subject's dMRI data (ΔGS). The table reports single‐ and two‐fiber agreement rates (AR) and angular differences (AD), along with apparent fiber density (AFD) difference, angular correlation coefficient (ACC), and generalized fractional anisotropy (GFA) difference. Better consistency is indicated by higher AR and ACC, and lower AD, AFD difference, and GFA difference. $n_{m}$ represents the number of measurements in each split dataset.

For AFD differences, SS3T‐CSD achieves significantly lower values (0.066) compared to MSMT‐CSD (0.206 for dHCP, 0.117 for BCP), indicating better amplitude consistency between split‐dataset reconstructions. The ACC shows high overall fODF similarity, with MSMT‐CSD achieving the highest ACC values (0.960 for dHCP, 0.927 for BCP) compared to SS3T‐CSD (0.916). GFA differences follow the same trend as ACC, with dHCP modeled with MSMT‐CSD benefiting the most from the high‐angular sampling scheme (150 measurements).

For all experiments, metrics related to voxels with 3‐fibers are not reported because of their low consistency (ARs: 26.0%–43.6% compared to 45%–92% for 1–2 fiber populations), confirming the known limitations of current dMRI approaches for complex crossing fiber configurations (Schilling et al. 2018), especially in early development when the anisotropy is low (Dubois et al. 2014).

To further quantify the consistency between these two reference methods, we conducted a direct comparison by applying both MSMT‐CSD and SS3T‐CSD reconstructions to the same dHCP subjects. The ACC between the two methods averages 84.5%±3.0%. The agreement in peak number detection shows substantial variation by fiber configuration: Single‐fiber voxels show 45.6%±7.4% agreement, while two‐fiber voxels show only 13.5%±4.4% agreement. ADs between corresponding peaks average $7.2^{{}^{\circ}}\pm 0.1^{{}^{\circ}}$ for single fibers and $12.7^{{}^{\circ}}\pm 0.1^{{}^{\circ}}$ for two‐fiber populations. These differences confirm that the two reconstruction methods capture fundamentally different aspects of the underlying microstructure, with SS3T‐CSD demonstrating higher sensitivity to crossing fiber configurations and MSMT‐CSD providing more conservative estimates with higher single‐fiber stability.

It is important to note that the consistency values reported in this section represent the variability inherent in classical reconstruction methods when applied to subsampled data. Deep learning models trained on complete datasets may achieve ARs that exceed these split‐dataset consistency values, as they learn from full diffusion acquisitions and can leverage spatial context to compensate for reconstruction inconsistencies.

### 4.2 Intra‐Site Performance

**FIGURE 2:**
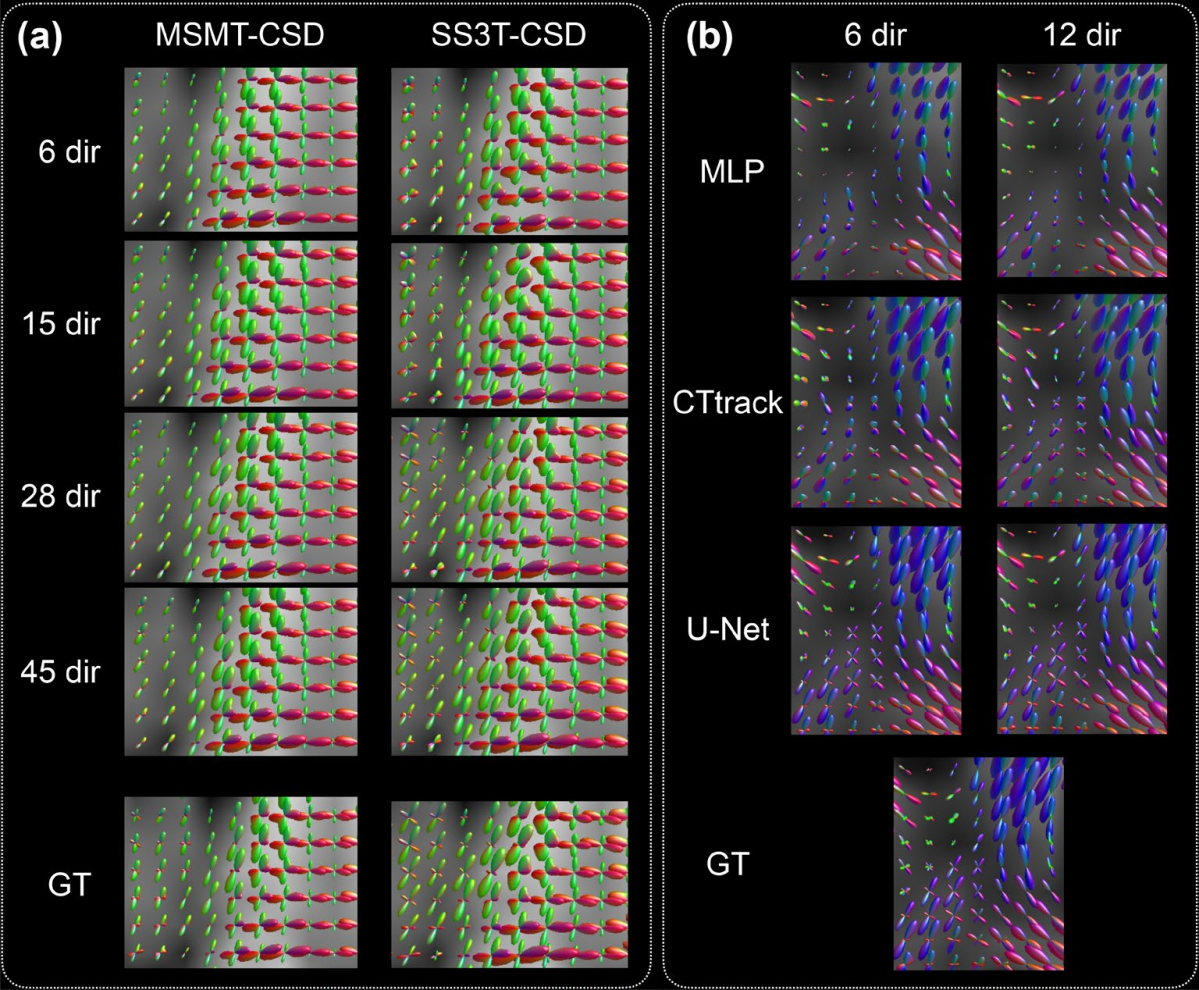
Qualitative examples of fiber orientation distribution function (fODF) estimation. (a) dHCP dataset: U‐Net predictions trained and tested using 6, 15, 28, and 45 diffusion gradient directions as input, with models trained using MSMT‐CSD (left) and SS3T‐CSD (right) reference reconstructions as ground truth. U‐Net is shown to demonstrate the effect of varying input directions on reconstruction quality for both ground truth types. (b) BCP dataset: MLP, CTtrack, and U‐Net predictions trained and tested using 6 and 12 diffusion gradient directions as input, with models trained using MSMT‐CSD reference reconstructions as ground truth. All three architectures are shown for comparison, while SS3T‐CSD is not included due to insufficient b1000 measurements (12) in BCP. Bottom rows show the corresponding ground truth (GT) reconstructions. Each model is trained on the respective number of input directions and evaluated on test subjects using the same input configuration. fODF glyphs are color‐coded by fiber orientation, visualized using MRtrix (Tournier et al. 2012).

Figure 2 provides qualitative examples, showing representative fODF reconstructions from each model architecture across different input configurations. For the dHCP dataset (Panel a), U‐Net predictions trained with varying numbers of input directions (6, 15, 28, 45) are compared against both MSMT‐CSD and SS3T‐CSD ground truth reconstructions. For the BCP dataset (Panel b), all three model architectures (MLP, CTtrack, U‐Net) are compared using 6 and 12 input directions.

#### 4.2.1 Performance on the dHCP Dataset

**FIGURE 3:**
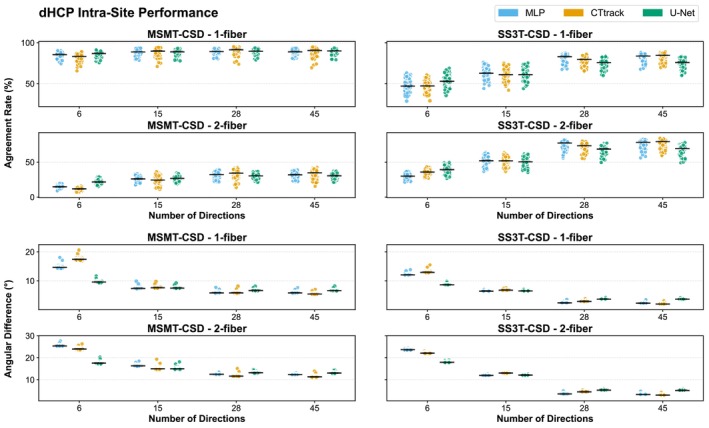
Model performance comparison for intra‐site evaluation on dHCP dataset. Agreement rates (top panels) and angular differences (bottom panels) between predicted and reference fODFs for MSMT‐CSD (left) and SS3T‐CSD (right) ground truth reconstructions. Results shown for three deep learning architectures (MLP, CTrack, U‐Net) across different input direction counts (6, 15, 28, 45), with separate analysis for single‐fiber (1‐fiber) and two‐fiber (2‐fiber) populations. SS3T‐CSD experiments are conducted only on dHCP data due to insufficient b1000 directions (only 12) in BCP. Higher agreement rates and lower angular differences indicate better performance. Each point represents individual test subjects, with horizontal lines showing median values.

We generally observe (Figure 3) similar performances of the different models for the AR and the AD when the number of input diffusion gradient directions is 15 or higher. For six directions, U‐Net models outperform in both metrics (AR and AD) for both MSMT‐ and SS3T‐CSD reference reconstructions serving as training GT. Statistical analysis using Wilcoxon signed–rank tests confirmed that U‐Net significantly outperformed both MLP and CTtrack architectures across multiple metrics. For example, at 6 directions with MSMT‐CSD ground truth, U‐Net showed significantly higher ARs for both single‐fiber (p<0.001 vs. both alternatives) and two‐fiber populations (p<0.001). Similar significant improvements were observed for AD (p<0.001), AFD difference (p<0.001), ACC (p<0.001), and GFA difference (p<0.001) across all tested direction counts. This can be explained by the large field of view (i.e., patches of 16^3^) of that method that compensates for the small number of directions. This trend is reversed for higher input directions, where CTtrack and the MLP score are slightly higher, especially for SS3T‐CSD.

**FIGURE 4:**
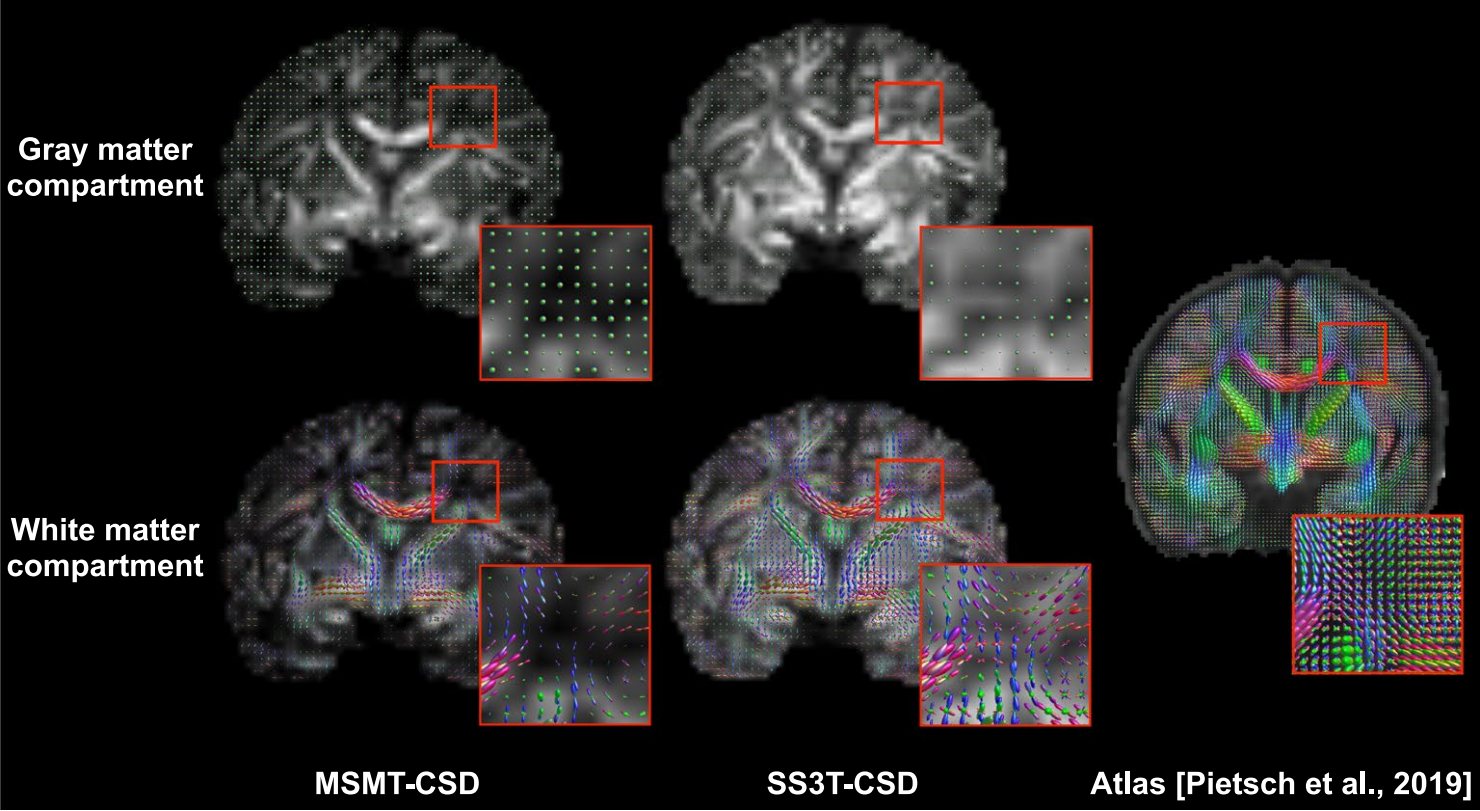
Qualitative comparison of coronal slices of the fODFs used as training GT for the learning‐based models, reconstructed with MSMT‐CSD and SS3T‐CSD, respectively, from the dMRI scan of a subject at 40 weeks PMA. The visualization presents both gray and white matter compartments overlaid on fractional anisotropy (FA) maps derived from the dMRI data. The reconstructions were generated and visualized using MRtrix (Tournier et al. 2012) and its fork MRtrix3Tissue (https://3Tissue.github.io). For anatomical reference, the rightmost image shows the corresponding slice from the brain atlas published by Pietsch et al. (2019). Inset boxes highlight detailed regions of the reconstructions, demonstrating the fiber orientation patterns in both compartments.

Interestingly, and as can be depicted qualitatively in Panel (a) of Figure 2 (shown for the case of U‐Net), 2‐fiber errors are significantly lower for SS3T‐CSD than MSMT‐CSD. This is consistent with the power of SS3T‐CSD in depicting crossing fibers compared to MSMT‐CSD in newborns (Dhollander et al. 2019). This can be observed in Figure 4, where crossing fibers between the cortico‐spinal tract and the corpus callosum are clearly delineated for SS3T‐CSD, where the gray matter component is not overestimated as in MSMT‐CSD. For anatomical reference, we include the corresponding slice from the atlas by Pietsch et al. (2019).

We also observe that all methods exhibit performance saturation when the number of input diffusion gradient directions increases from 28 to 45 in Figure 3. Performance improvements were statistically significant from 6 to 15 and from 15 to 28 directions for all methods (p<0.001), but showed divergent patterns from 28 to 45 directions, with most MSMT‐CSD configurations reaching a statistical plateau (p>0.05), while SS3T‐CSD maintained significant improvements across most metrics (p<0.001).

For the AFD difference (Figure S1), we make similar observations as for AR and AD regarding the edge of U‐Net compared to other models for 6 directions, especially for SS3T‐CSD. However, this edge is kept for all input direction configurations. The ACC analysis (Figure S2) shows U‐Net achieving the highest values across most configurations, reaching approximately 0.90 for 6 directions with SS3T‐CSD ground truth compared to 0.83–0.85 for MLP and CTtrack. Similarly, GFA difference analysis (Figure S3) reveals U‐Net achieving the lowest GFA differences at 6 directions across both ground truth methods, demonstrating superior anisotropy preservation in a low‐data scenario.

#### 4.2.2 Performance on the BCP Dataset

**FIGURE 5:**
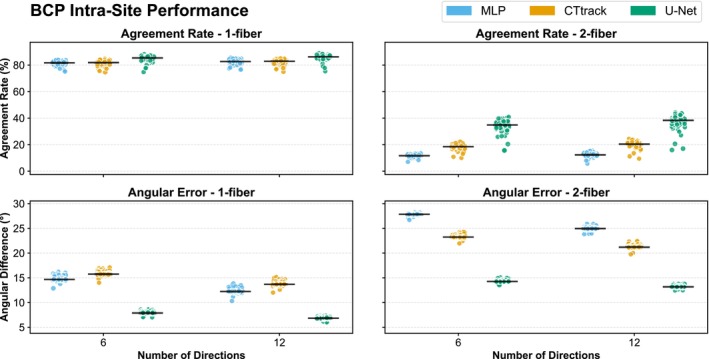
Model performance comparison for intra‐site evaluation on BCP dataset using MSMT‐CSD ground truth. Agreement rates (top) and angular differences (bottom) between predicted and reference fODFs for single‐fiber (left) and two‐fiber (right) populations. Results shown for three deep learning architectures (MLP, CTtrack, U‐Net) with 6 and 12 input diffusion directions. The BCP dataset contains older subjects (2–24 months) compared to dHCP neonates. Each point represents individual test subjects, with horizontal lines showing median values.

We observe (Figure 5) a superior performance of U‐Net compared to the other methods, which is more pronounced for 2‐fibers, for both AR in the number of peaks and their AD, as also qualitatively shown in Figure 2 (right column). These performance differences were statistically significant (Wilcoxon signed–rank test, p<0.001). For instance, at 6 directions, U‐Net showed significantly higher ARs and lower angular errors for both single‐fiber and two‐fiber populations compared to MLP and CTtrack (all p<0.001). A similar observation can be made for the AFD difference (Figure S1, right panel). Moreover, we notice a significant improvement when going from 6 to 12 directions (p<0.001). ACC analysis (Figure S2, right panel) shows U‐Net reaching ~0.96 versus ~0.90 for other methods, whereas GFA differences (Figure S3) remain consistently lowest for U‐Net.

### 4.3 Impact of Age‐Related Domain Shift in dHCP

Our analysis included 1008 statistical tests examining both between‐group differences (Mann–Whitney *U*) and training effects (Wilcoxon signed–rank) across all age configurations and metrics.

**FIGURE 6:**
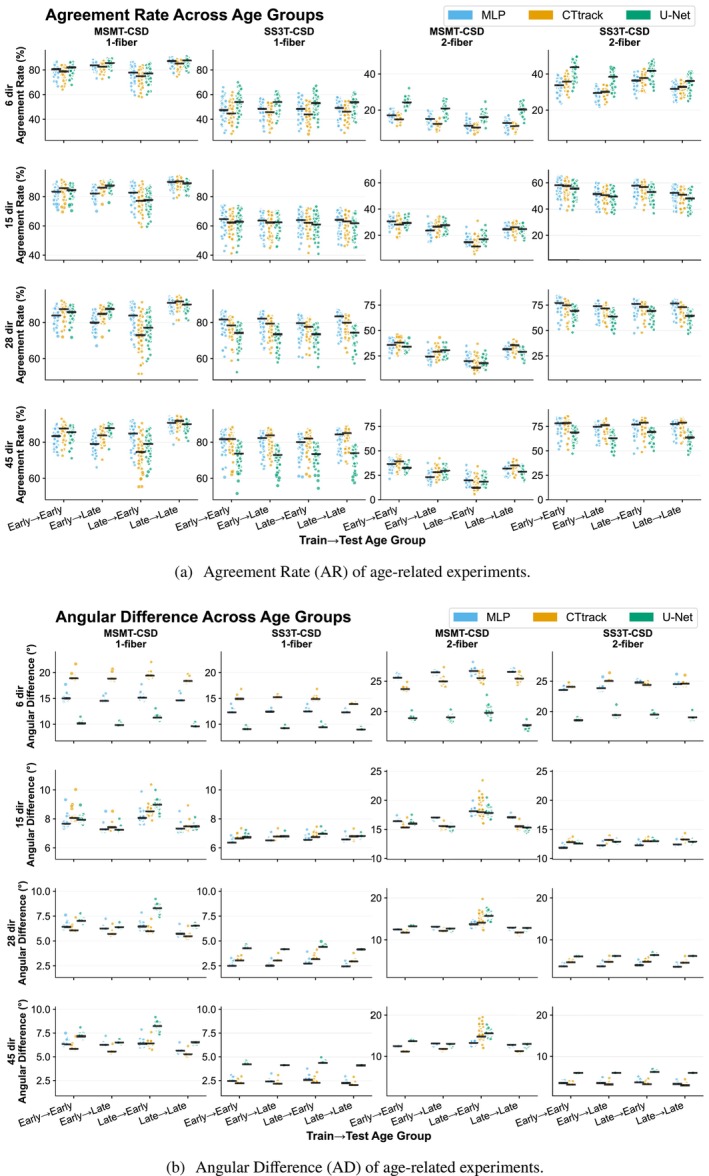
Age‐related domain shift analysis within dHCP dataset (33–45 weeks of age). Agreement rates (Panel a) and angular differences (Panel b) between predicted and reference fODFs across four experimental conditions: early → early (train/test on 33–38 weeks), late → late (train/test on 41–45 weeks), early → late (train on early, test on late), and late → early (train on late, test on early). Results shown for MSMT‐CSD and SS3T‐CSD ground truth with varying input directions (6, 15, 28, 45) and all three architectures (MLP, CTtrack, U‐Net). SS3T‐CSD experiments are conducted only on dHCP data due to insufficient b1000 directions (only 12) in BCP. Each point represents individual test subjects, with horizontal lines showing median values. Single‐fiber (1‐fiber) and two‐fiber (2‐fiber) populations demonstrate different sensitivities to age‐related domain shifts.

The AR (Figure 6a) and AD (Figure 6b) analyses reveal asymmetric age‐related domain shifts. Training on late‐stage subjects (41–45 weeks) and testing on early subjects (33–38 weeks) shows minimal performance degradation, while the reverse direction (early → late) exhibits substantial domain shift, particularly for two‐fiber populations. This can be observed especially for 2‐fiber populations. Age‐related domain shifts showed significant effects across all methods and metrics (Mann–Whitney *U* test, p<0.001). Models trained and tested on matched age groups (early → early, late → late) consistently outperformed cross‐age scenarios (early → late, late → early), with early‐to‐late generalization showing more performance degradation than late‐to‐early cross‐age transfer (p<0.001), confirming the presence of age‐related domain shifts even within the narrow postmenstrual age range studied. SS3T‐CSD demonstrates greater robustness to these age‐related variations compared to MSMT‐CSD across all experimental configurations.

Regarding model performance, U‐Net maintains superiority with 6 input directions across all age configurations, whereas MLP and CTtrack perform better at higher numbers of directions (28–45), especially on the SS3T‐CSD GT. Notably, MLP shows continued improvement from 28 to 45 directions, particularly with SS3T‐CSD ground truth, unlike the other models, which plateau. The AFD analysis (Figure S4) confirms these patterns. The early → late transfer shows significantly higher AFD errors compared to other age configurations, while SS3T‐CSD maintains lower domain shift sensitivity similar to the AR and AD metrics. Additional ACC and GFA analyses in Figures S5 and S6 further confirm SS3T‐CSD GT's superior robustness, maintaining stable ACC and GFA values across all age transfers.

### 4.4 Evaluating Domain Shift Mitigation

Domain adaptation effectiveness was evaluated through 2268 statistical tests: 1512 comparing adaptation strategies and target subject progressions, plus 756 direct method‐to‐method comparisons validating architecture‐specific performance.

Across both transfer directions, domain adaptation strategies demonstrated high effectiveness. Statistical analysis confirmed that MoM achieved significant improvements compared to baseline (no adaptation) in 325 out of 336 configurations (96.7%) and fine‐tuning showed significant improvements compared to baseline in 324 out of 336 configurations (96.4%) across all three architectures (Wilcoxon signed–rank test, p<0.05).

#### 4.4.1 Trained on dHCP and Tested on BCP

**FIGURE 7:**
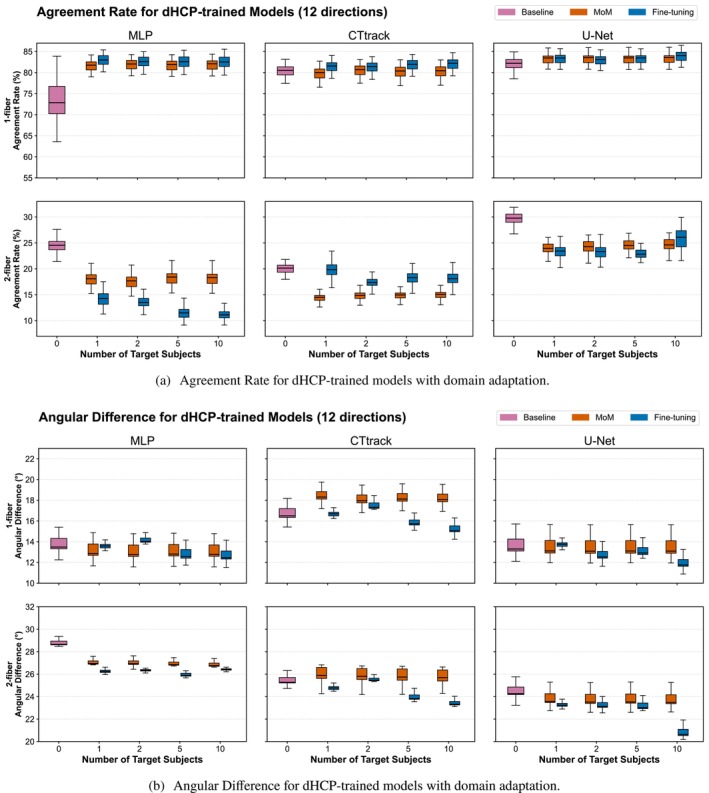
Domain adaptation performance for dHCP‐trained models tested on BCP target domain. Agreement rates (Panel a) and angular differences (Panel b) between predicted and reference fODFs using increasing numbers of BCP target subjects (1, 2, 5, 10) for adaptation. Results compare three adaptation strategies: baseline (no adaptation), Method of Moments (MoM) harmonization, and fine‐tuning across all three architectures (MLP, CTtrack, U‐Net) with 12 input directions. Box plots show distribution across test subjects, representing cross‐dataset transfer from neonatal (dHCP, 33–45 weeks of age) to baby (BCP, 2–24 months postnatal) populations. Single‐fiber (1‐fiber) and two‐fiber (2‐fiber) populations demonstrate different adaptation characteristics (6‐direction results are provided in Figure S8).

We generally observe (Figure 7) that the single fiber configuration does not benefit as much as the 2‐fiber configuration from an increased number of target subjects for domain shift attenuation for all models and both MoM and fine‐tuning. Similarly, fine‐tuning benefits more from the number of target subjects compared to MoM. Moreover, in the 2‐fibers configuration, the more target subjects we add, the more fine‐tuning overperforms MoM. For dHCP → BCP transfer, statistical analysis confirmed MoM effectiveness in 164 out of 168 configurations (97.6%) and fine‐tuning effectiveness in 160 out of 168 configurations (95.2%) across all three architectures (Wilcoxon signed–rank test, p<0.05). For single fiber populations, MoM is slightly better or equal to fine‐tuning in performance, especially for the AD metric. Except for the single‐fiber configuration in AR where MLP is overperforming other methods, we observe that U‐Net is generally the best model in domain shift attenuation for both MoM and fine‐tuning (Wilcoxon signed–rank test: U‐Net significantly outperformed MLP and CTtrack with p<0.001 across 89% of all comparisons; specifically for angular error (97% wins), AR in 2‐fiber populations (99% wins), ACC (97% wins), GFA (93% wins), and AFD (92% wins)). When we go from 6 to 12 input diffusion gradient directions, no noticeable trends were observed except the global performance improvement with the MLP benefiting the most from the increase in input samples (6‐direction results are provided in Figures S8, S10, and S11).

For AFD difference (Figure S7), fine‐tuning always surpasses MoM, with no significant improvement with the number of target subjects. This suggests that with a single subject, the distribution of the target AFD can be learned.

These findings are further confirmed by complementary quantitative analyses of amplitude and anisotropy characteristics (Figure 9). The ACC analysis (Figure 9a) reveals that fine‐tuning achieves substantially higher global fODF similarity compared to both baseline and MoM approaches, with ACC values increasing from baseline ranges of 0.75–0.80 to above 0.80 for all models, and U‐Net approaching 0.90 (for 6 directions). The GFA error also shows a similar pattern where fine‐tuning generally surpasses the MoM, with U‐Net consistently achieving the lowest GFA differences and lowest standard deviations, and benefiting most from fine‐tuning, demonstrating that its spatial context modeling is particularly effective during domain transfer.

#### 4.4.2 Trained on BCP and Tested on dHCP

We similarly observe that fine‐tuning benefits more from increasing the number of target subjects compared to MoM for the AD and the AR. This can be particularly observed in AD. However, this increase is not very pronounced and only starts to show at 10 subjects (p<0.05 for improvement from 5 to 10 subjects). This can be particularly observed for U‐Net in the case of 2‐fiber populations. Fine‐tuning generally outperforms the MoM, but not in all cases. For instance, for AR and 2‐fiber populations, MLP performs generally better with MoM compared to fine‐tuning, and U‐Net is better for the case of the number of target subjects is five or fewer. U‐Net is generally the best model for addressing domain shifts (Wilcoxon signed–rank test: p<0.001 across multiple metrics; angular error 90% wins, AR 2‐fiber 89% wins, ACC 97% wins, GFA 86% wins), and it improves more with the number of target subjects and less with the number of gradient directions, the opposite of MLP and CTtrack. Statistical analysis confirmed MoM effectiveness in 161 out of 168 configurations (95.8%) and fine‐tuning effectiveness in 164 out of 168 configurations (97.6%) for BCP → dHCP transfer across all three architectures (Wilcoxon signed–rank test, p<0.05).

**FIGURE 8:**
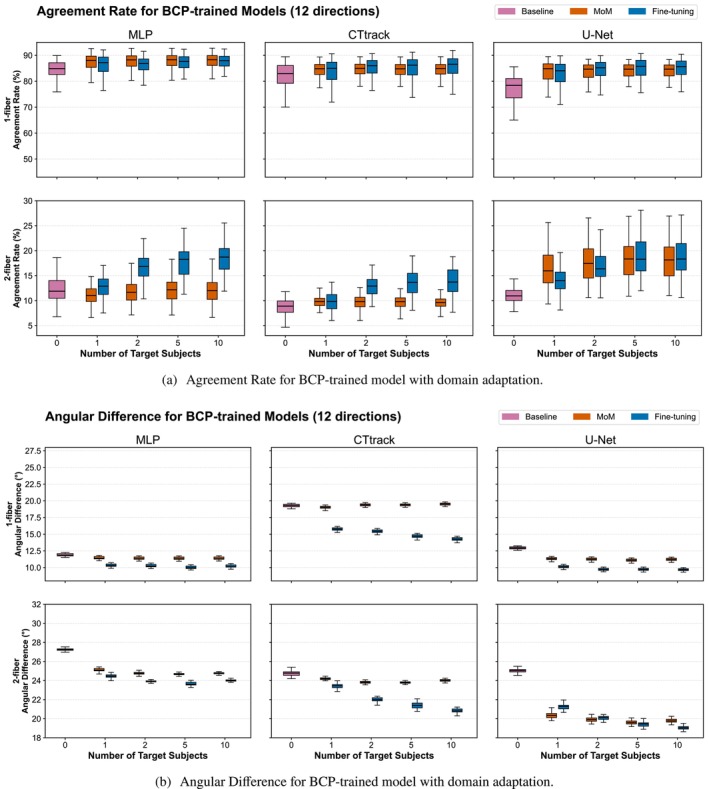
Domain adaptation performance for BCP‐trained models tested on dHCP target domain. Agreement rates (Panel a) and angular differences (Panel b) between predicted and reference fODFs using increasing numbers of dHCP target subjects (1, 2, 5, 10) for adaptation. Results compare three adaptation strategies: baseline (no adaptation), Method of Moments (MoM) harmonization, and fine‐tuning across all three architectures (MLP, CTtrack, U‐Net) with 12 input directions. Box plots show distribution across test subjects, representing cross‐dataset transfer from baby (BCP, 2–24 months postnatal) to neonatal (dHCP, 33–45 weeks of age) populations. Single‐fiber (1‐fiber) and two‐fiber (2‐fiber) populations demonstrate different adaptation characteristics (6‐direction results are provided in Figure S9).

**FIGURE 9:**
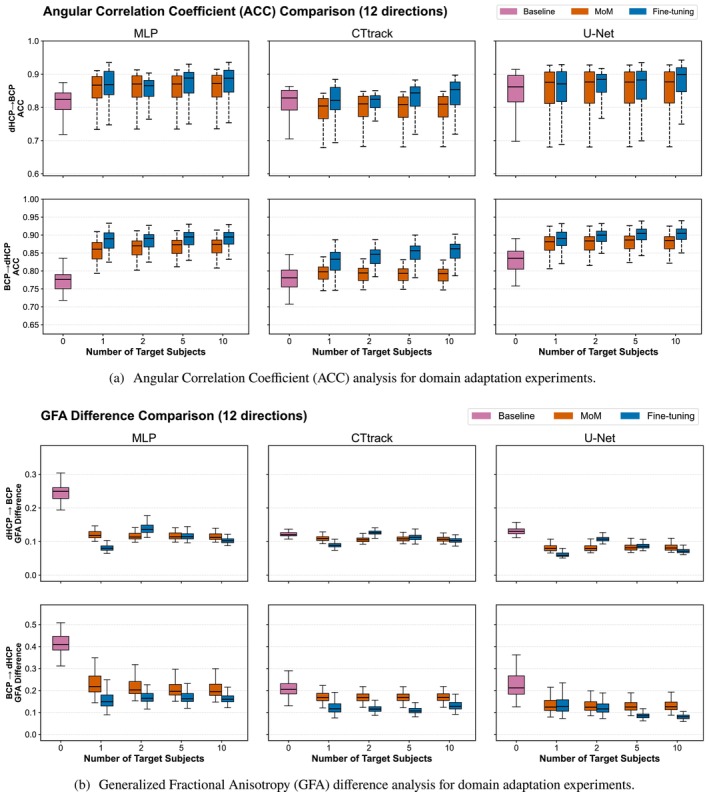
Quantitative metrics analysis for domain adaptation experiments. ACC values (Panel a) computed as correlation between predicted and reference fiber orientations and GFA differences (Panel b) computed as absolute difference between predicted and reference anisotropy values for dHCP‐trained models tested on BCP target domain (left panels) and BCP‐trained models tested on dHCP target domain (right panels). Results compare three adaptation strategies: baseline (no adaptation), Method of Moments (MoM) harmonization, and fine‐tuning using increasing numbers of target domain subjects (1, 2, 5, 10) across all three architectures (MLP, CTtrack, U‐Net) with 12 input directions. Box plots show distribution across test subjects, representing cross‐dataset transfer between neonatal and baby populations. Fine‐tuning consistently achieves higher ACC values compared to baseline and MoM, with U‐Net demonstrating the most stable fiber orientation correlations across domain adaptation scenarios. U‐Net consistently achieves the lowest GFA differences and benefits most from fine‐tuning (6‐direction results are provided in Figures S10 and S11).

For AFD difference (Figure S7, Panel b), fine‐tuning always surpasses MoM, with no significant improvement with the number of target subjects, up to five subjects as observed for AR and AD. The overall domain adaptation patterns for AR and AD in the BCP‐to‐dHCP transfer are illustrated in Figure 8, with complementary ACC and GFA analyses shown in Figure 9a,b (6‐direction results are provided in Figures S9–S11).

The ACC analysis reveals that BCP → dHCP transfer presents substantially greater challenges for maintaining fiber orientation correlations compared to the reverse direction. Baseline ACC values are lower (0.74–0.79, for 6 directions) compared to dHCP → BCP transfer (0.77–0.83, for 6 directions), reflecting the biological complexity of predicting neonatal brain microstructure from baby‐trained models. Fine‐tuning provides critical improvements, elevating ACC values to 0.78–0.88 across all architectures. U‐Net demonstrates the most stable performance with the highest ACC values, consistently increasing with the number of subjects in fine‐tuning, followed by MLP and finally CTtrack.

GFA difference analysis further emphasizes the challenge of the BCP → dHCP domain shift. Baseline GFA differences are dramatically elevated, reaching 0.40–0.45 for MLP and 0.20–0.25 for CTtrack and U‐Net, indicating substantial anisotropy estimation errors when applying baby‐trained models to neonatal data. Fine‐tuning provides essential corrections, reducing GFA differences to 0.15–0.20 for MLP and 0.08–0.15 for U‐Net, though these remain higher than the reverse transfer direction. This pattern reflects the inherent difficulty in predicting the rapid microstructural changes occurring during early brain development, where neonatal tissue properties differ substantially from the more mature patterns captured in baby training data as also demonstrated previously (Lin, Gholipour, et al. 2024). The consistent superiority of U‐Net across both ACC and GFA metrics reinforces its robustness for challenging domain adaptation scenarios involving developmental populations.

## 5 Discussion

In this study, we extensively investigated the performance and robustness of different deep‐learning models on dMRI‐derived fODFs. We conducted intra‐site experiments on two datasets, the developing dHCP and the BCP, and inter‐site experiments to evaluate domain shift attenuation techniques. Specifically, we examined the effect of the number of input diffusion gradient directions, the influence of different ground truth configurations (MSMT‐ and SS3T‐CSD), and age domain shift on model performance and employed two domain adaptation strategies: the MoM and fine‐tuning.

### 5.1 Architecture‐Specific Performance

Our intra‐site experiments revealed that U‐Net consistently outperformed the other models when fewer diffusion directions were used, particularly with the dHCP dataset and the SS3T‐CSD ground truth. However, with an increased number of directions, MLP and CTtrack showed marginally but statistically significantly better performance.

The intra‐site experiments also show a plateau in the performance observed across all models from 28 to 45 directions, suggesting diminishing returns beyond a certain threshold of input directions and hence, a reduction in scanning time compared to non‐deep‐learning models. This important finding has direct implications for clinical scanning protocols, as it indicates potential for significant scan time reduction without compromising fODF reconstruction quality. For SS3T‐CSD in the same intra‐site experimental settings, this performance is acceptable for both single‐ and two‐fiber cases, reaching around 75% in AR and around 3° in the AD.

In the intra‐site BCP experiments, U‐Net maintained its edge, particularly for 2‐fiber populations, with less noticeable improvement when increasing from 6 to 12 directions. This could be attributed to the more consistent anatomical structures in older infants compared to the neonatal cohort of dHCP. The robustness of U‐Net's performance is also reflected in ACC and GFA metrics, particularly in low‐direction regimes where spatial context modeling proves most valuable.

### 5.2 Impact of Reference Reconstruction Methods

While architectural differences explain much of the performance variation, the choice of reference reconstruction method proved to be also influential across all architectures: Using SS3T‐CSD as the reference reconstruction method yielded statistically significantly lower ADs for 2‐fiber populations, with a more pronounced improvement with the number of input measurements, highlighting its efficacy in capturing crossing fibers compared to MSMT‐CSD in developing brains (Dhollander et al. 2019). This is also confirmed by the lower ground‐truth consistency in MSMT‐CSD compared to SS3T‐CSD in crossing fibers, despite the former benefiting from more diffusion measurements.

These findings underscore a fundamental limitation: The performance of DL‐based fODF estimation models is bounded by the quality and consistency of the data, in particular crossing fiber voxels. Challenges faced by current dMRI models, including MSMT‐CSD and SS3T‐CSD, in accurately estimating multiple fiber populations and low angular crossing fibers have been shown by Schilling et al. (2018), and also in our algorithm consistency analysis comparison experiment. In fact, the performance saturation at around 28 input directions is likely not a neural network limit but a model‐data limit. The more diverse and consistent the data, the more crossing fibers and crossing angles can be resolved, even for as few as 6 directions, as shown in our experiments, bypassing theoretical limits.

These inherent limitations of CSD‐based reference reconstructions also explain why direct comparison with unsupervised methods would be methodologically problematic. Our supervised models are explicitly trained to reproduce CSD reconstructions, while unsupervised methods learn alternative representations that may better capture the underlying fiber architecture.

### 5.3 Domain Shift Characteristics and Mitigation Strategies

Age‐related experiments in dHCP demonstrated reduced domain shifts when training on later age groups, with SS3T‐CSD proving more robust to age‐related variability than MSMT‐CSD. The consistent statistical significance of age‐related effects (p<0.001) within narrow age windows demonstrates that even subtle developmental changes create measurable domain shifts, with practical implications for age‐specific model calibration in clinical deployment.

Importantly, the magnitude of these age‐related domain shifts within dHCP (33–45 weeks PMA) is substantially smaller than the inter‐site domain shifts, reflecting the distinction between subtle biological developmental changes and more substantial biological and technical domain shifts. Our inter‐site experiments (dHCP vs. BCP) actually encompass much larger age‐related variations, as they span from neonates (33–45 weeks PMA) to babies (2–24 months), with differences in scanners and acquisition protocols. This combination of biological and technical domain shifts in the inter‐site scenario represents the most challenging and practically relevant deployment conditions. The substantial performance degradation observed in these cross‐site scenarios necessitated the evaluation of practical mitigation strategies. In fact, our results highlighted the complexity of estimating neonatal brain microstructure from baby‐trained models, more than the reverse direction. For domain shift attenuation, fine‐tuning generally surpassed MoM, particularly as the number of target subjects increased. U‐Net emerged as the most reliable model for domain adaptation across different scenarios, with its performance benefiting more from an increase in target subjects than from additional gradient directions. For most of the configurations, fine‐tuning with five target subjects yielded satisfactory results, as also demonstrated in other applications such as semantic segmentation (Lhermitte et al. 2024; Zalevskyi et al. 2025). The choice of limited target subjects (1–10) reflects both established practices in medical imaging domain adaptation (Ghafoorian et al. 2017; Valverde et al. 2019), where effective adaptation has been demonstrated with minimal target data, and practical constraints in clinical deployment scenarios where acquiring sufficient dMRI data for reliable CSD‐based reference reconstruction is time‐consuming and resource‐intensive (Calamuneri et al. 2018; Golkov et al. 2016; Tournier et al. 2013). This, however, depends on the consistency and variability of the few target subjects. Future work can focus on optimizing the choice of these target subjects and how synthetic data can help compensate for real data.

### 5.4 Limitations and Future Directions

It is important to note that obtaining absolute *ground truth* fiber orientations remains an open challenge in the field, as it would require extensive histological validation, which is rarely available, especially in developing brains. Classical reconstruction methods (Dhollander and Connelly 2016; Jeurissen et al. 2014; Tournier et al. 2004) serve as practical reference standards, despite being approximations of the underlying anatomy. These methods, based on physical models and mathematical constraints, provide reasonable estimates that have been validated through various indirect means, including anatomical studies and phantom experiments.

Future work can also attempt to merge reference fODF reconstruction algorithms in a way that leverages each of their advantages and potentially overcomes individual method constraints. Additionally, extending domain adaptation approaches to cross‐shell scenarios where models trained on one *b*‐value are deployed on datasets with different *b*‐values represents a promising research direction, as prior work has highlighted both the challenges of multi‐shell *b*‐value generalizability (Nath, Lyu, et al. 2019) and the complexity of cross‐shell transformations (Dugan and Carmichael 2023), though such approaches would require specialized frameworks to handle the distinct microstructural sensitivities captured by different *b*‐values and the associated cross‐shell domain shifts. The practical challenges of limited target data availability could be addressed through the optimized selection of target subjects for fine‐tuning and exploration of how synthetic data generation can help compensate for real data limitations. Future work could also explore controlled studies that systematically disentangle biological developmental changes from technical domain shifts in larger pediatric cohorts.

Domain adaptation is a promising strategy to foster model generalization in medical imaging, and many methods have been proposed recently (Guan and Liu 2021) and increase the likelihood of potential deployment in clinical settings. Some of these challenges in fODF estimation, linked to the spatial resolution, for example, have been attempted to be addressed recently by implicit neural representations (Dwedari et al. 2024), and others related to the broad heterogeneity of the input acquisition (gradient directions, *b*‐values, scanner type, etc.) by Ewert et al. (2024) using an encoder‐decoder framework to learn latent representations of the signal and the *b*‐vectors. The convergence of these diverse methodological advances with our findings on architecture‐specific robustness and practical domain adaptation strategies strengthens the path toward reliable clinical deployment.

## 6 Conclusion

Although we employed well‐established deep learning architectures (MLP, CNN‐Transformer, and U‐Net), our contribution lies in the rigorous evaluation of their robustness under realistic clinical deployment scenarios. The systematic domain shift analysis revealed architecture‐specific properties that were previously unknown, demonstrating that spatial context models (U‐Net) excel with limited data while direct mapping approaches (MLP) benefit more from increased input dimensionality. These findings have direct implications for clinical practice, where scanning protocols, patient populations, and hardware configurations vary significantly across institutions.

Overall, our study highlights the potential of deep learning for fODF estimation of developing brains while underscoring key challenges related to domain shifts. While domain adaptation techniques like MoM and fine‐tuning offer promising solutions, further research is needed to refine these methods, particularly in selecting optimal target subjects and improving ground‐truth models used to generate the GT fODFs. Ultimately, improving data consistency and model robustness will be crucial for translating these models into real‐world clinical applications.

## Ethics Statement

This retrospective research study used open‐source human subject data from the Developing Human Connectome Project and the Baby Connectome Project, respectively, where ethical approval was not required per the data licenses.

## Conflicts of Interest

The authors declare no conflicts of interest.

## Supporting information


**Data S1:** hbm70367‐sup‐0001‐Figures.pdf.

## Data Availability

The analyzed datasets were publicly available: Developing Human Connectome Project (dHCP): https://www.developingconnectome.org/data‐release/third‐data‐release/. Baby Connectome Project (BCP): https://www.humanconnectome.org/study/lifespan‐baby‐connectome‐project/. The code is publicly available at https://github.com/Medical‐Image‐Analysis‐Laboratory/dl_fiber_domain_shift.

## Endnotes

^1^

https://www.developingconnectome.org/data‐release/third‐data‐release/.

^2^

https://www.humanconnectome.org/study/lifespan‐baby‐connectome‐project/overview.
