# Supplementary material for: Deep Learning for fODF Estimation in Infant Brains: Model Comparison, Ground‐Truth Impact, and Domain Shift Mitigation
Source: Hum Brain Mapp. 2025 Oct 7;46(14):e70367. doi: 10.1002/hbm.70367 (PMC12501774; doi:10.1002/hbm.70367)
Supplement: Supplementary file 1 — Data S1: hbm70367‐sup‐0001‐Figures.pdf. [file HBM-46-e70367-s001.pdf]

Supplementary Materials

Intra-site performance analysis

- Figure S1: Apparent Fiber Density (AFD) difference analysis for intra-site evaluation, demonstrating U-Net's consistent superiority in amplitude preservation across all configurations.
- Figure S2: Angular Correlation Coefficient (ACC) analysis for intra-site evaluation, complementing the agreement rate and angular difference findings presented in the main paper.
- Figure S3: Generalized Fractional Anisotropy (GFA) difference analysis for intra-site evaluation, showing U-Net's superior anisotropy preservation in low-data regimes.

Age-related domain shift analysis

- Figure S4: AFD difference analysis for age-related domain shifts within dHCP dataset, complementing the age-related patterns shown in the main paper.
- Figure S5: ACC analysis for age-related domain shifts within dHCP dataset, providing additional validation of age-related robustness patterns.
- Figure S6: GFA difference analysis for age-related domain shifts within dHCP dataset, supporting developmental robustness findings.

Inter-site domain adaptation analysis

- Figure S7: AFD difference analysis for domain adaptation experiments, showing the differential challenges of dHCP$\rightarrow$BCP versus BCP$\rightarrow$dHCP transfer.
- Figure S8: Agreement rate and angular difference analysis for dHCP-trained models with 6 gradient directions, demonstrating model robustness under severe angular undersampling conditions when tested on BCP target domain.
- Figure S9: Agreement rate and angular difference analysis for BCP-trained models with 6 gradient directions, illustrating the increased challenge of BCP$\rightarrow$dHCP transfer with limited gradient directions.
- Figure S10: ACC analysis for domain adaptation experiments with 6 gradient directions, highlighting U-Net's effectiveness in maintaining high correlations even with severe angular undersampling.
- Figure S11: GFA difference analysis for domain adaptation experiments with 6 gradient directions, showing anisotropy preservation under minimal gradient direction conditions.

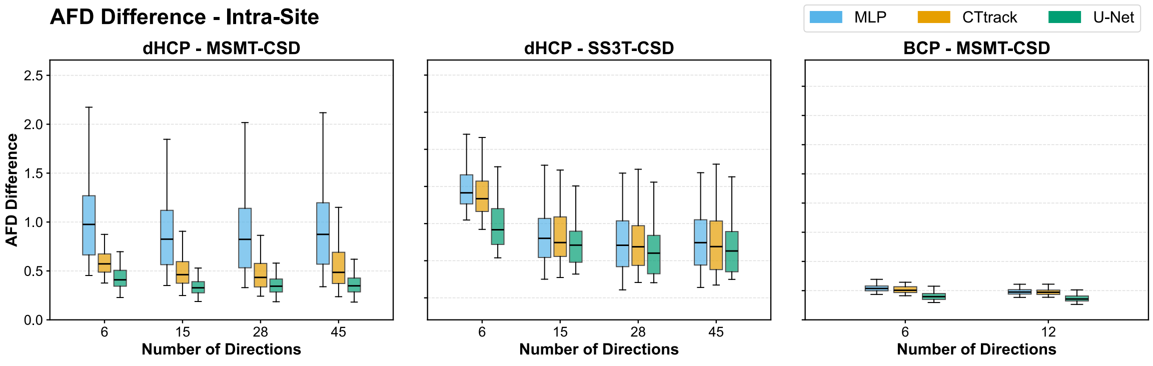

Figure S1. Apparent Fiber Density (AFD) difference analysis for intra-site evaluation. U-Net consistently achieves the lowest AFD differences across all configurations, demonstrating superior amplitude preservation. AFD differences computed as Mean Absolute Percentage Difference (MAPD) between predicted and reference fODF amplitudes across dHCP dataset with MSMT-CSD and SS3T-CSD ground truth (6, 15, 28, 45 input directions) and BCP dataset with MSMT-CSD ground truth (6, 12 input directions). SS3T-CSD experiments are conducted only on dHCP data due to insufficient b1000 directions (only 12) in BCP. Results shown for all three architectures (MLP, CTtrack, U-Net) with box plots displaying distribution across test subjects.

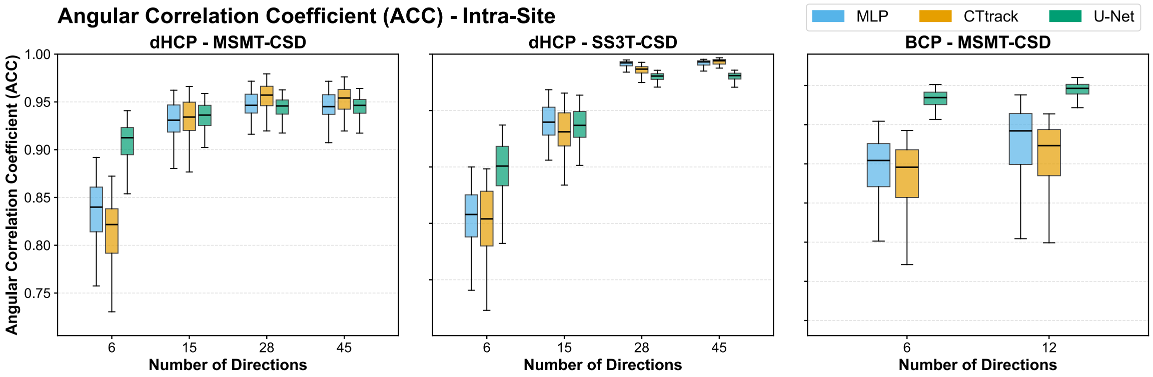

Figure S2. **Angular Correlation Coefficient (ACC) analysis for intra-site evaluation.** These results complement the agreement rate and angular difference findings presented in the main paper (Figures 3 and 5). ACC values computed as correlation between predicted and reference fiber orientations across dHCP dataset with MSMT-CSD and SS3T-CSD ground truth (6, 15, 28, 45 input directions) and BCP dataset with MSMT-CSD ground truth (6, 12 input directions). SS3T-CSD experiments are conducted only on dHCP data due to insufficient b1000 directions (only 12) in BCP. Results shown for all three architectures (MLP, CTtrack, U-Net) with box plots displaying distribution across test subjects. Higher ACC values indicate better preservation of fiber orientation relationships, with SS3T-CSD demonstrating superior performance for low direction counts, particularly evident in the 6-direction configuration, where U-Net achieves ~0.90 correlation.

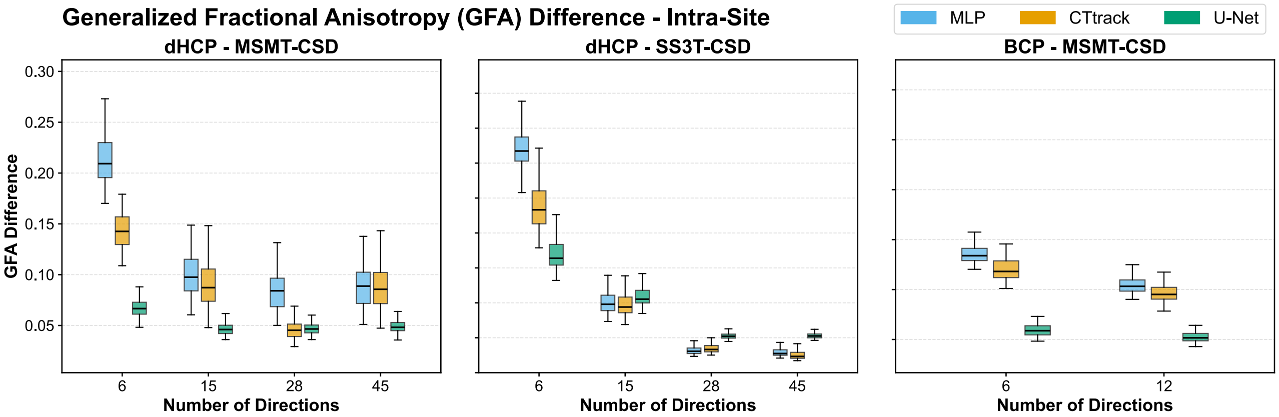

Figure S3. **Generalized Fractional Anisotropy (GFA) difference analysis for intra-site evaluation.** U-Net achieves the lowest GFA differences at 6 directions across all datasets, demonstrating superior anisotropy preservation in low-data regimes. GFA differences computed as absolute difference between predicted and reference anisotropy values across dHCP dataset with MSMT-CSD and SS3T-CSD ground truth (6, 15, 28, 45 input directions) and BCP dataset with MSMT-CSD ground truth (6, 12 input directions). SS3T-CSD experiments are conducted only on dHCP data due to insufficient b1000 directions (only 12) in BCP. Results shown for all three architectures (MLP, CTrack, U-Net) with box plots displaying distribution across test subjects. Lower GFA differences indicate better preservation of tissue anisotropy characteristics, with performance differences between methods becoming less pronounced at higher direction counts.

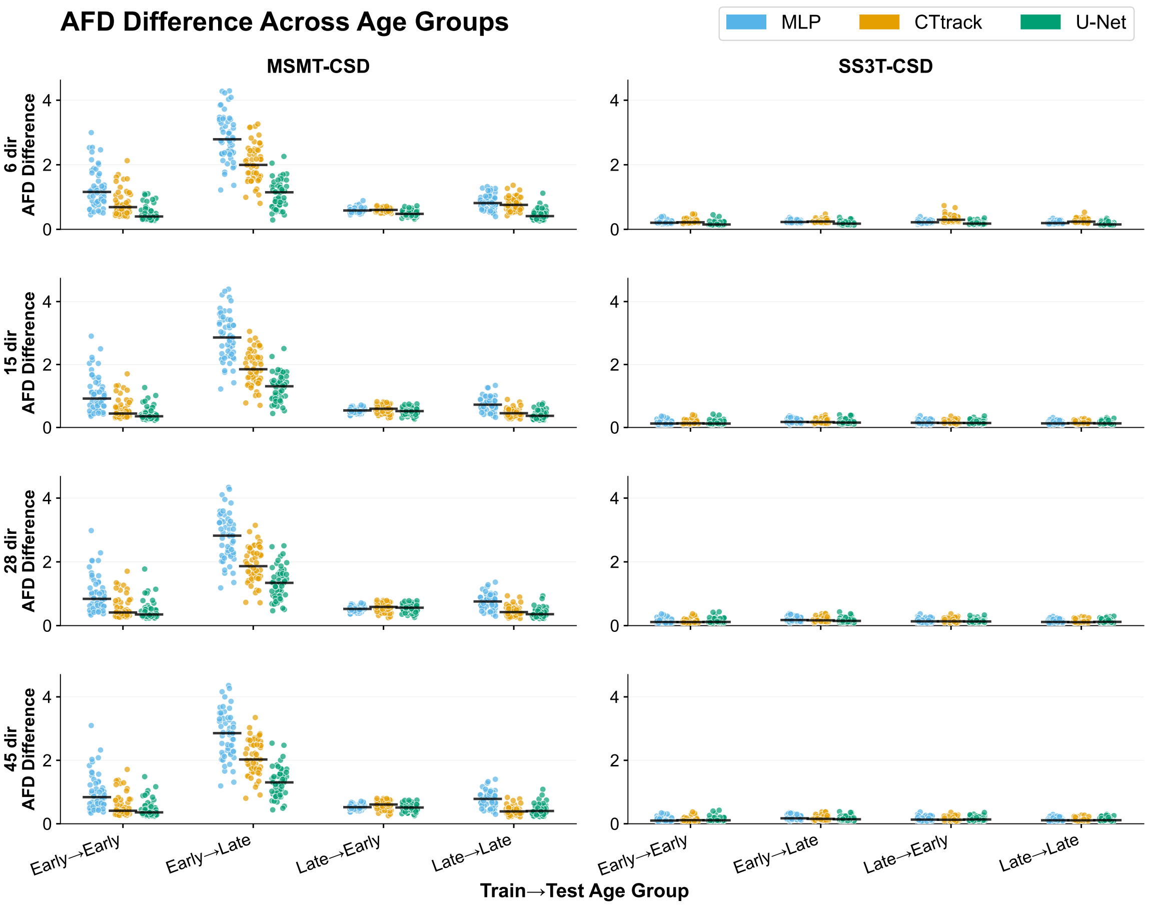

Figure S4. Age-related domain shift analysis for Apparent Fiber Density (AFD) difference within dHCP dataset (33–45 weeks age). These results complement the age-related agreement rate and angular difference patterns shown in main paper (Figure 6). AFD differences computed as Mean Absolute Percentage Difference (MAPD) between predicted and reference fODF amplitudes across four experimental conditions: Early→Early (train/test on 33–38 weeks), Late→Late (train/test on 41–45 weeks), Early→Late (train on early, test on late), and Late→Early (train on late, test on early). Results shown for MSMT-CSD and SS3T-CSD ground truth with varying input directions (6, 15, 28, 45) and all three architectures (MLP, CTtrack, U-Net). SS3T-CSD experiments are conducted only on dHCP data due to insufficient b1000 directions (only 12) in BCP. Each point represents individual test subjects, with horizontal lines showing median values.

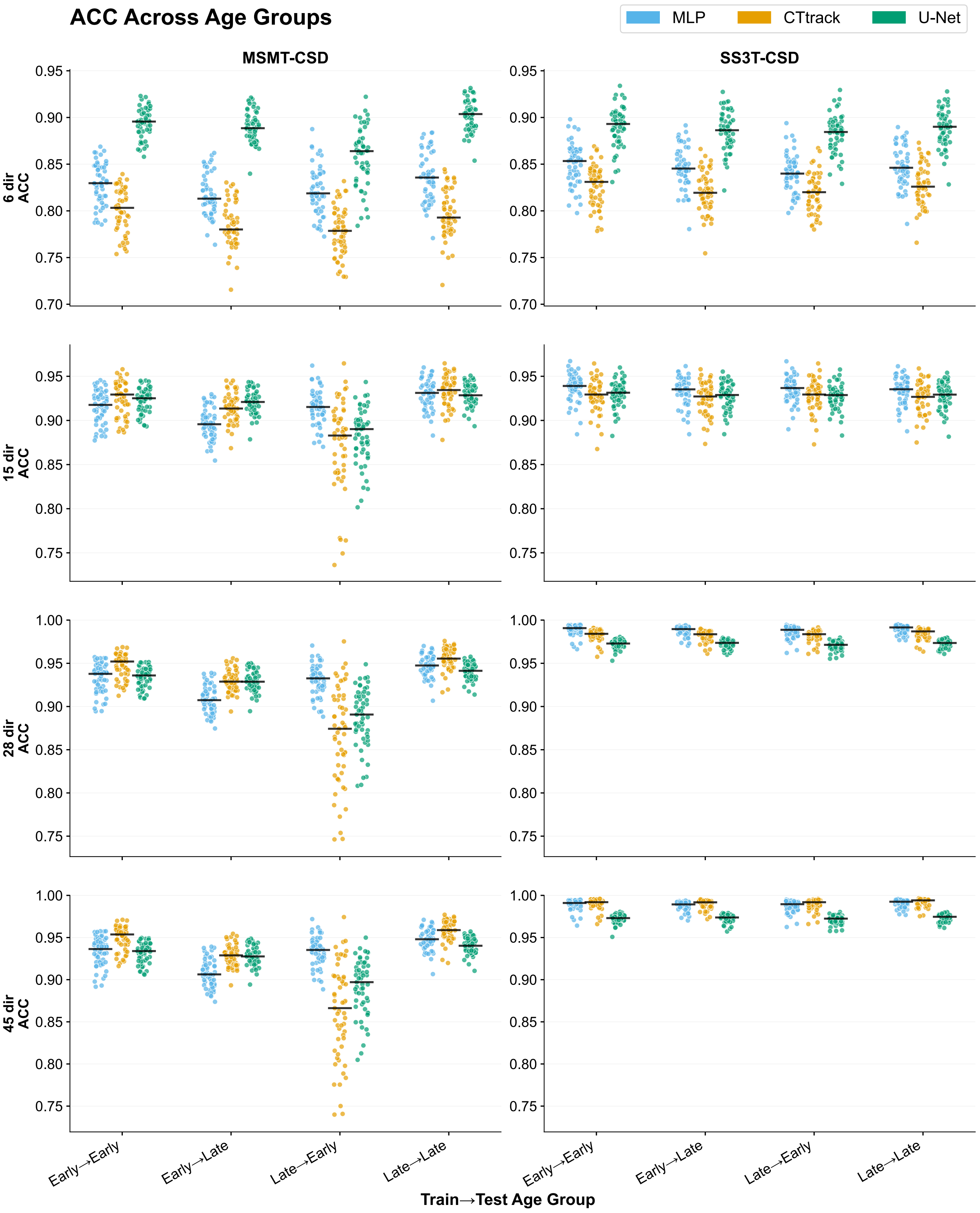

Figure S5. Angular Correlation Coefficient (ACC) analysis for age-related domain shift within dHCP dataset. These findings provide additional validation of the age-related robustness patterns demonstrated in main paper (Figure 6). ACC values computed as correlation between predicted and reference fiber orientations across four experimental conditions: Early→Early (train/test on 33–38 weeks), Late→Late (train/test on 41–45 weeks), Early→Late (train on early, test on late), and Late→Early (train on late, test on early). Results shown for MSMT-CSD and SS3T-CSD ground truth with varying input directions (6, 15, 28, 45) and all three architectures (MLP, CTtrack, U-Net). SS3T-CSD experiments are conducted only on dHCP data due to insufficient b1000 directions (only 12) in BCP. Each point represents individual test subjects, with horizontal lines showing median values. SS3T-CSD shows greater robustness to age-related domain shifts, particularly evident in the 6-direction condition, where performance remains more stable across age transitions.

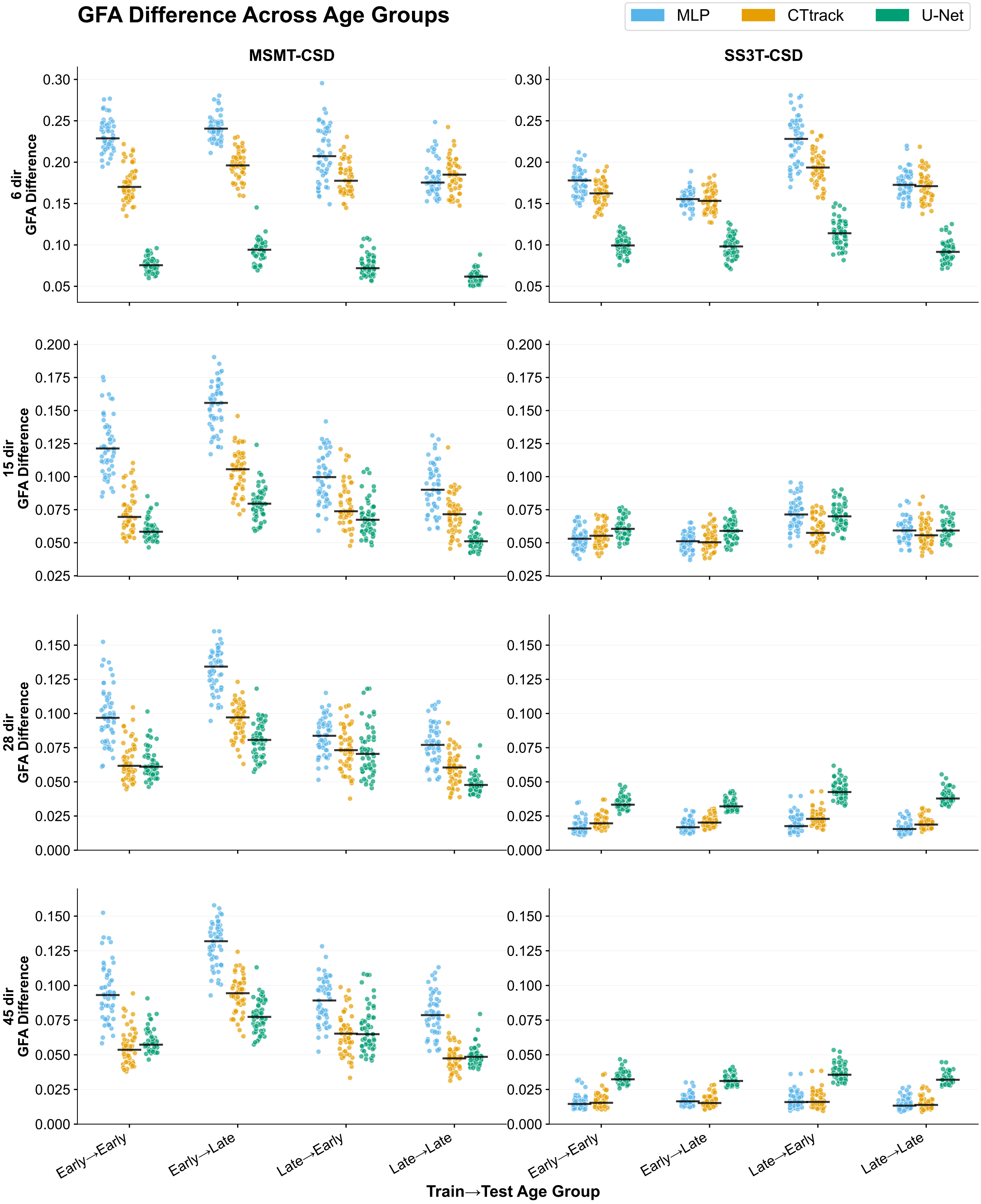

Figure S6. **Generalized Fractional Anisotropy (GFA) difference analysis for age-related domain shift within dHCP dataset.** These anisotropy preservation results further support the developmental robustness findings from the main paper (Figure 6). GFA differences computed as absolute difference between predicted and reference anisotropy values across four experimental conditions: Early→Early (train/test on 33–38 weeks), Late→Late (train/test on 41–45 weeks), Early→Late (train on early, test on late), and Late→Early (train on late, test on early). Results shown for MSMT-CSD and SS3T-CSD ground truth with varying input directions (6, 15, 28, 45) and all three architectures (MLP, CTtrack, U-Net). SS3T-CSD experiments are conducted only on dHCP data due to insufficient b1000 directions (only 12) in BCP. Each point represents individual test subjects, with horizontal lines showing median values. U-Net demonstrates superior anisotropy preservation across all age-related domain shift scenarios, with SS3T-CSD showing reduced sensitivity to developmental changes compared to MSMT-CSD.

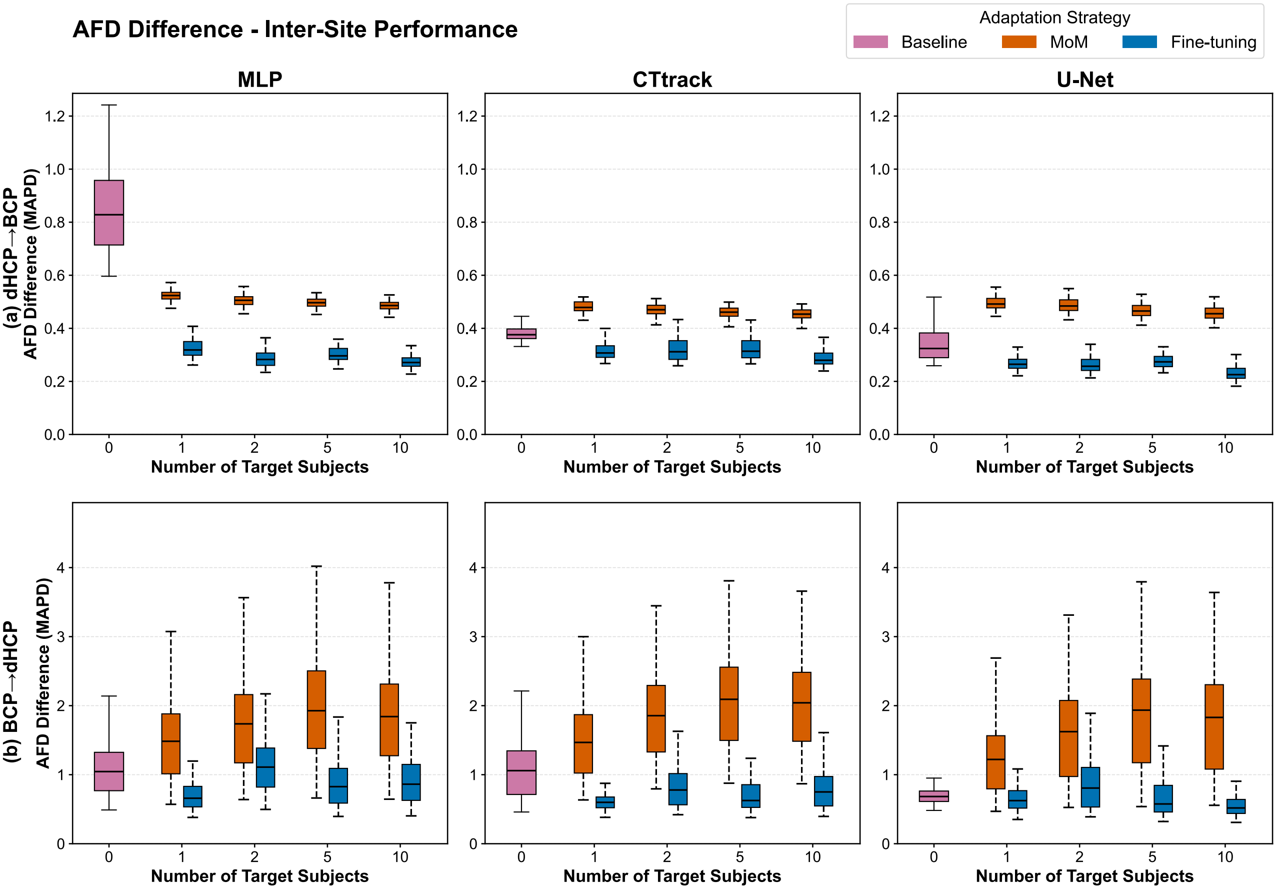

Figure S7. **Apparent Fiber Density (AFD) difference analysis for domain adaptation experiments.** BCP→dHCP transfer (panel b) presents substantially greater challenges than dHCP→BCP transfer (panel a), reflecting the biological complexity of predicting neonatal brain patterns from mature baby data. AFD differences computed as Mean Absolute Percentage Difference (MAPD) between predicted and reference fODF amplitudes for dHCP-trained models tested on BCP target domain (panel a) and BCP-trained models tested on dHCP target domain (panel b). Results compare three adaptation strategies: baseline (no adaptation), Method of Moments (MoM) harmonization, and fine-tuning using increasing numbers of target domain subjects (1, 2, 5, 10) across all three architectures (MLP, CTtrack, U-Net) with 6 and 12 input directions. Box plots show distribution across test subjects, representing cross-dataset transfer between neonatal and baby populations.

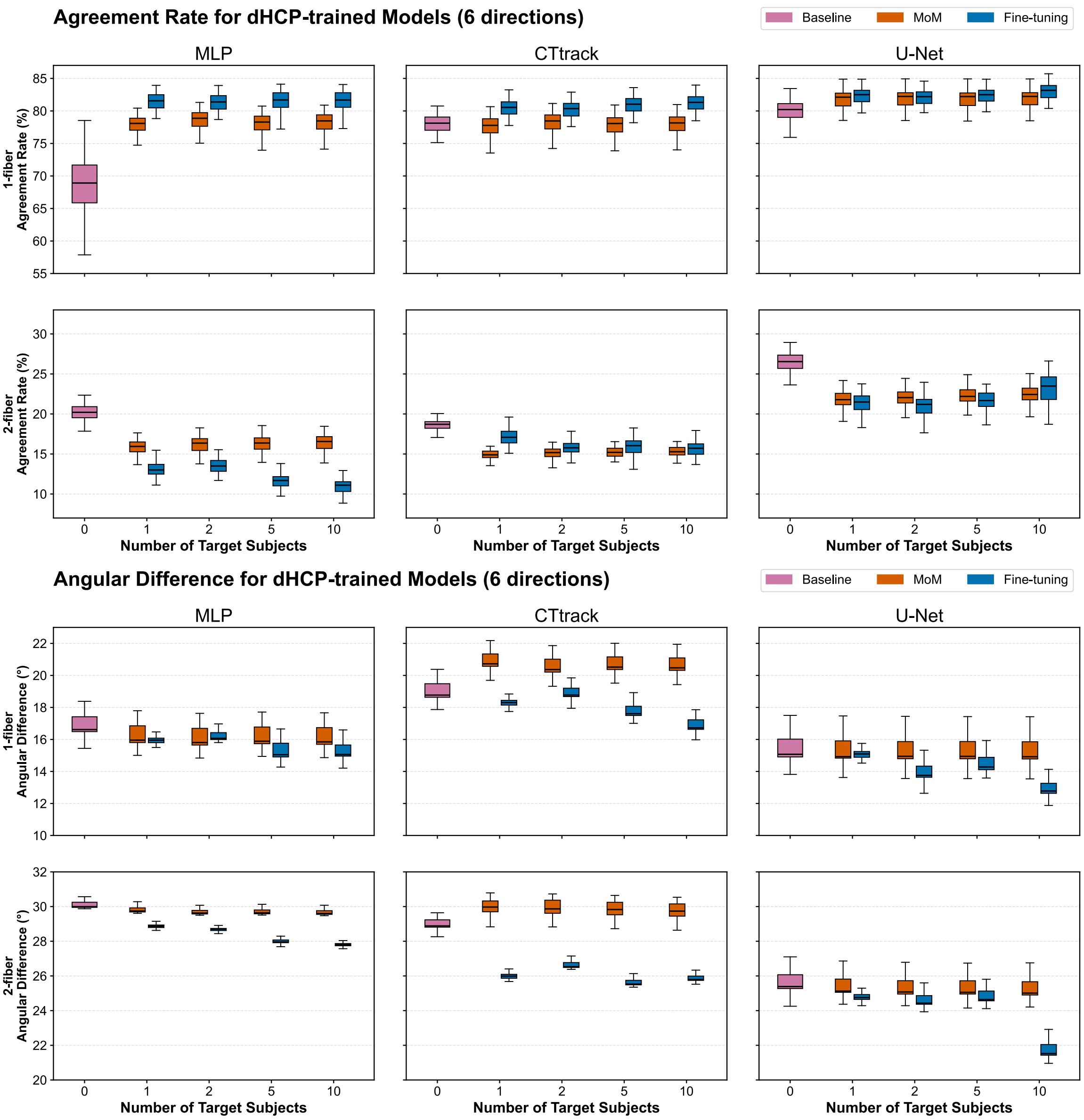

Figure S8. Domain adaptation performance for dHCP-trained models with 6 gradient directions. Agreement rates and angular differences for models tested on BCP target domain using only 6 input directions. Despite severe angular undersampling, U-Net maintains superior performance through effective spatial context utilization, achieving reasonable domain adaptation even with limited data. These results complement the 12-direction analysis presented in Figure 7.

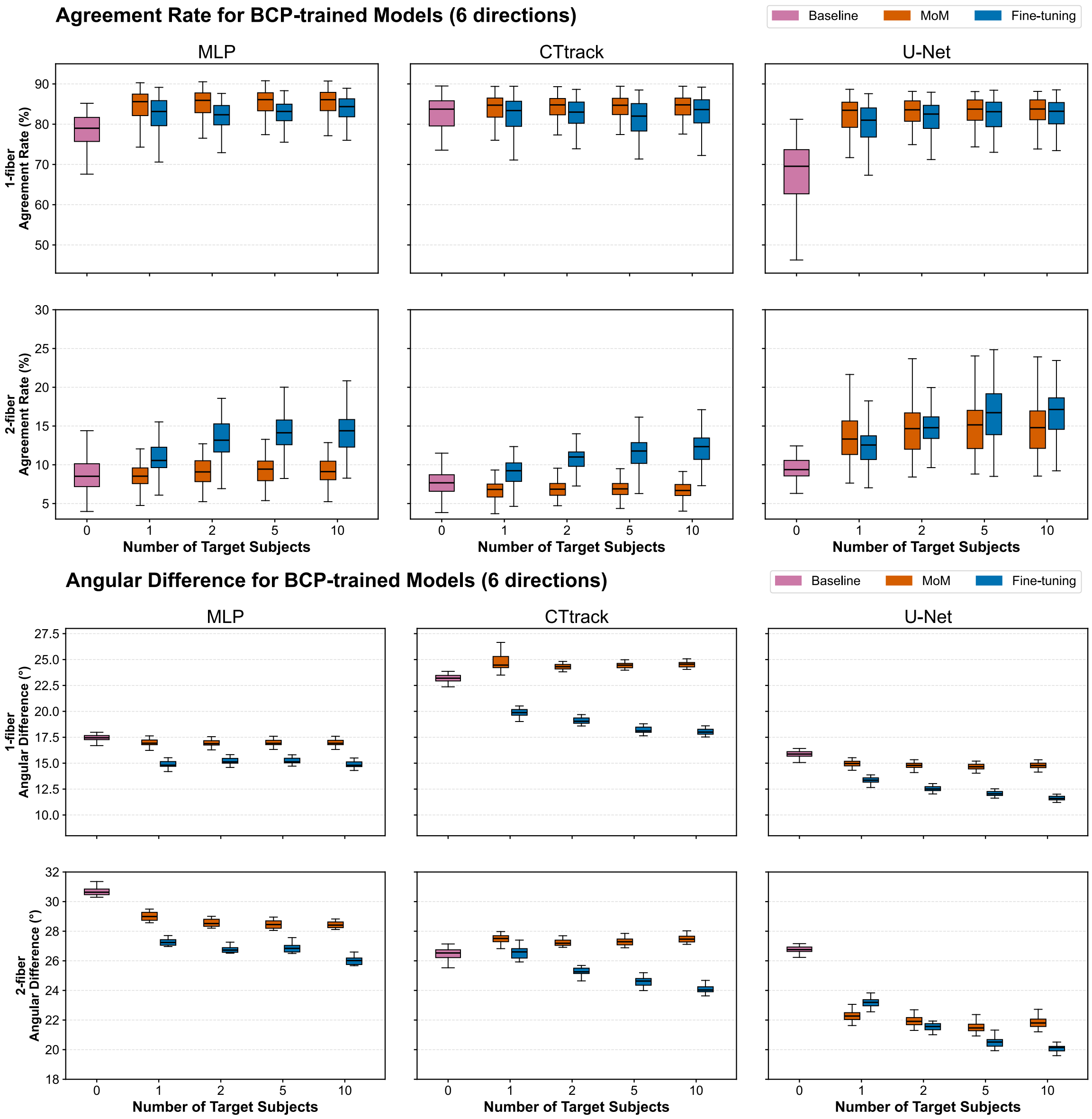

Figure S9. Domain adaptation performance for BCP-trained models with 6 gradient directions. Agreement rates and angular differences for models tested on dHCP target domain using only 6 input directions. The increased challenge of BCP→dHCP transfer is particularly evident with limited gradient directions. These results complement the 12-direction analysis presented in Figure 8.

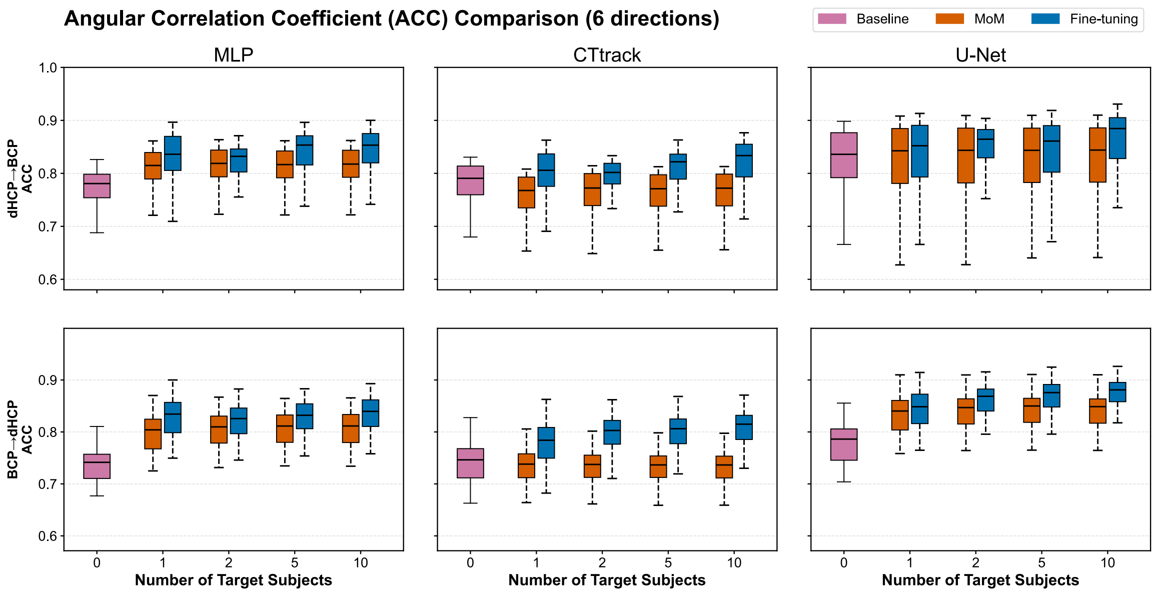

Figure S10. ACC analysis for domain adaptation with 6 gradient directions. Angular correlation coefficient values demonstrate that U-Net achieves remarkably high correlations even with severe angular undersampling, highlighting the effectiveness of spatial context modeling for limited-data scenarios. These results complement the 12-direction analysis presented in Figure 9a.

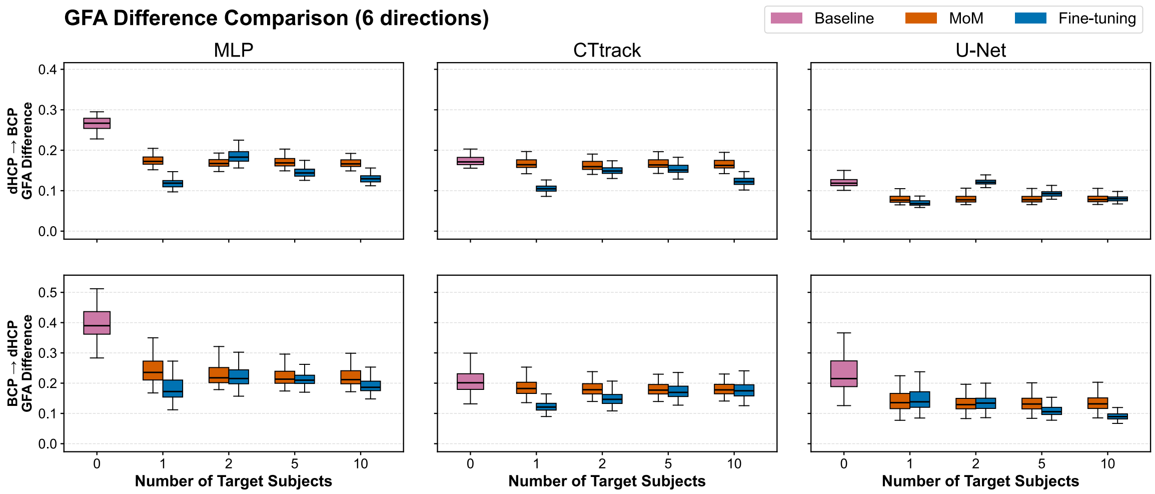

Figure S11. GFA difference analysis for domain adaptation with 6 gradient directions. Anisotropy preservation under severe angular undersampling conditions, showing that fine-tuning remains effective even with minimal gradient directions. These results complement the 12-direction analysis presented in Figure 9b.
